# Targeting Neurons with Functional Oxytocin Receptors: A Novel Set of Simple Knock-In Mouse Lines for Oxytocin Receptor Visualization and Manipulation

**DOI:** 10.1523/ENEURO.0423-21.2022

**Published:** 2022-02-14

**Authors:** Yukiko U. Inoue, Hideki Miwa, Kei Hori, Ryosuke Kaneko, Yuki Morimoto, Eriko Koike, Junko Asami, Satoshi Kamijo, Mitsuhiko Yamada, Mikio Hoshino, Takayoshi Inoue

**Affiliations:** 1 Department of Biochemistry and Cellular Biology, National Institute of Neuroscience, National Center of Neurology and Psychiatry, Kodaira, Tokyo 187-8502, Japan; 2 Department of Neuropsychopharmacology, National Institute of Mental Health, National Center of Neurology and Psychiatry, Kodaira, Tokyo 187-8553, Japan; 3 KOKORO-Biology Group, Laboratories for Integrated Biology, Graduate School of Frontier Biosciences, Osaka University, Suita, Osaka 565-0871, Japan

**Keywords:** Cre driver mouse, genome editing, knock-in mice, oxytocin, oxytocin receptor, reporter mouse

## Abstract

The neuropeptide oxytocin (Oxt) plays important roles in modulating social behaviors. Oxt receptor (Oxtr) is abundantly expressed in the brain and its relationship to socio-behavioral controls has been extensively studied using mouse brains. Several genetic tools to visualize and/or manipulate Oxtr-expressing cells, such as fluorescent reporters and Cre recombinase drivers, have been generated by ES-cell based gene targeting or bacterial artificial chromosome (BAC) transgenesis. However, these mouse lines displayed some differences in their Oxtr expression profiles probably because of the complex context and integrity of their genomic configurations in each line. Here, we apply our sophisticated genome-editing techniques to the *Oxtr* locus, systematically generating a series of knock-in mouse lines, in which its endogenous transcriptional regulations are intactly preserved and evaluate their expression profiles to ensure the reliability of our new tools. We employ the epitope tagging strategy, with which C-terminally fused tags can be detected by highly specific antibodies, to successfully visualize the Oxtr protein distribution on the neural membrane with super-resolution imaging for the first time. By using T2A self-cleaving peptide sequences, we also induce proper expressions of tdTomato reporter, codon-improved Cre recombinase (iCre), and spatiotemporally inducible Cre-ERT2 in Oxtr-expressing neurons. Electrophysiological recordings from tdTomato-positive cells in the reporter mice support the validity of our tool design. Retro-orbital injections of AAV-PHP.eB vector into the Cre line further enabled visualization of recombinase activities in the appropriate brain regions. Moreover, the first-time Cre-ERT2 line drives Cre-mediated recombination in a spatiotemporally controlled manner on tamoxifen (TMX) administration. These tools thus provide an excellent resource for future functional studies in Oxt-responsive neurons and should prove of broad interest in the field.

## Significance Statements

Here we develop a novel series of genome-edited mouse lines to help understand the circuit mechanisms underlying oxytocin (Oxt) actions, by enabling the visualization and manipulation of Oxt receptor (Oxtr)-expressing neurons in a manner precisely reflecting their endogenous expression profiles. The epitope tagging strategy allows super-resolution imaging to decipher the 3-D distributions of Oxtr protein on neural membranes for the first time. The red fluorescent tdTomato reporter serves as a reliable visualization tool for Oxtr-expressing cells, whose expression profiles are validated using electrophysiological recordings. Both iCre and iCre-ERT2 drivers, which properly induce recombinase activities in Oxtr-expressing cells, would largely contribute to viral vector-dependent functional analyses and fate mapping studies. These tools thus offer a new option for the research communities to improve our knowledge of Oxt-Oxtr circuitry.

## Introduction

The neuropeptide oxytocin (Oxt) has attracted great attention not only from researchers but also from the general public because of its profound social (Froemke and Young, 2021), pro-social (Marsh et al., 2021), anxiolytic (Neumann and Slattery, 2016), and anti-stress (Onaka and Takayanagi, 2019) behavioral effects in animals. Thanks to the advanced neuro-technologies, including optogenetics (Deisseroth, 2011; Grinevich and Neumann, 2021), chemogenetics (Grund et al., 2019; Grinevich and Neumann, 2021), and calcium imaging (Hung et al., 2017; Resendez et al., 2020; Maldonado et al., 2021), the complex neurobiology of the Oxt system has been gradually revealed over the last decade. The Oxt receptor (Oxtr), a member of the G-protein-coupled receptor (GPCR) family, processes and transfers signals into the cytoplasm to modulate downstream circuitries (Grinevich and Neumann, 2021). Although a growing number of studies have shed light on Oxt-Oxtr signaling in various brain regions such as the prefrontal cortex (PFC; Nakajima et al., 2014; Tan et al., 2019), lateral septum (Menon et al., 2018; Horiai et al., 2020), nucleus accumbens (Dölen et al., 2013; Nardou et al., 2019), hippocampus (Owen et al., 2013; Raam et al., 2017), ventral tegmental area (Hung et al., 2017), and others, each study has used different tools to visualize and manipulate Oxtr-expressing neurons.

Some studies have used an antibody (called OXTR-2) generated in a research laboratory to detect Oxtr proteins (Marlin et al., 2015; Mitre et al., 2016). Unfortunately, this antibody is not available for the public to replicate the experimental findings. In addition, because of the general difficulty in generating antibodies to lipophilic GPCRs with low epitope exposures, no anti-Oxtr antibody suitable for immunohistochemistry is commercially available. From these reasons, the *Oxtr-Venus* knock-in mouse line was generated (Yoshida et al., 2009) and used for receptor visualization (Dölen et al., 2013; Hung et al., 2017; Newmaster et al., 2020). However, contrary to our expectations, expressions of the Venus reporter in the brain was reported to largely decrease during development (Newmaster et al., 2020). In this reporter line, the first coding exon had been replaced with Venus sequences (Extended Data Fig. 1-1*b*). The resulting knock-in allele lacks its endogenous configurations of intron and 3′ untranslated region (UTR) sequences, both of which likely contain transcriptional regulatory elements essential for the spatiotemporally coordinated Oxtr expressions. This might considerably affect the Venus reporter expression profiles.

**Figure 1.**
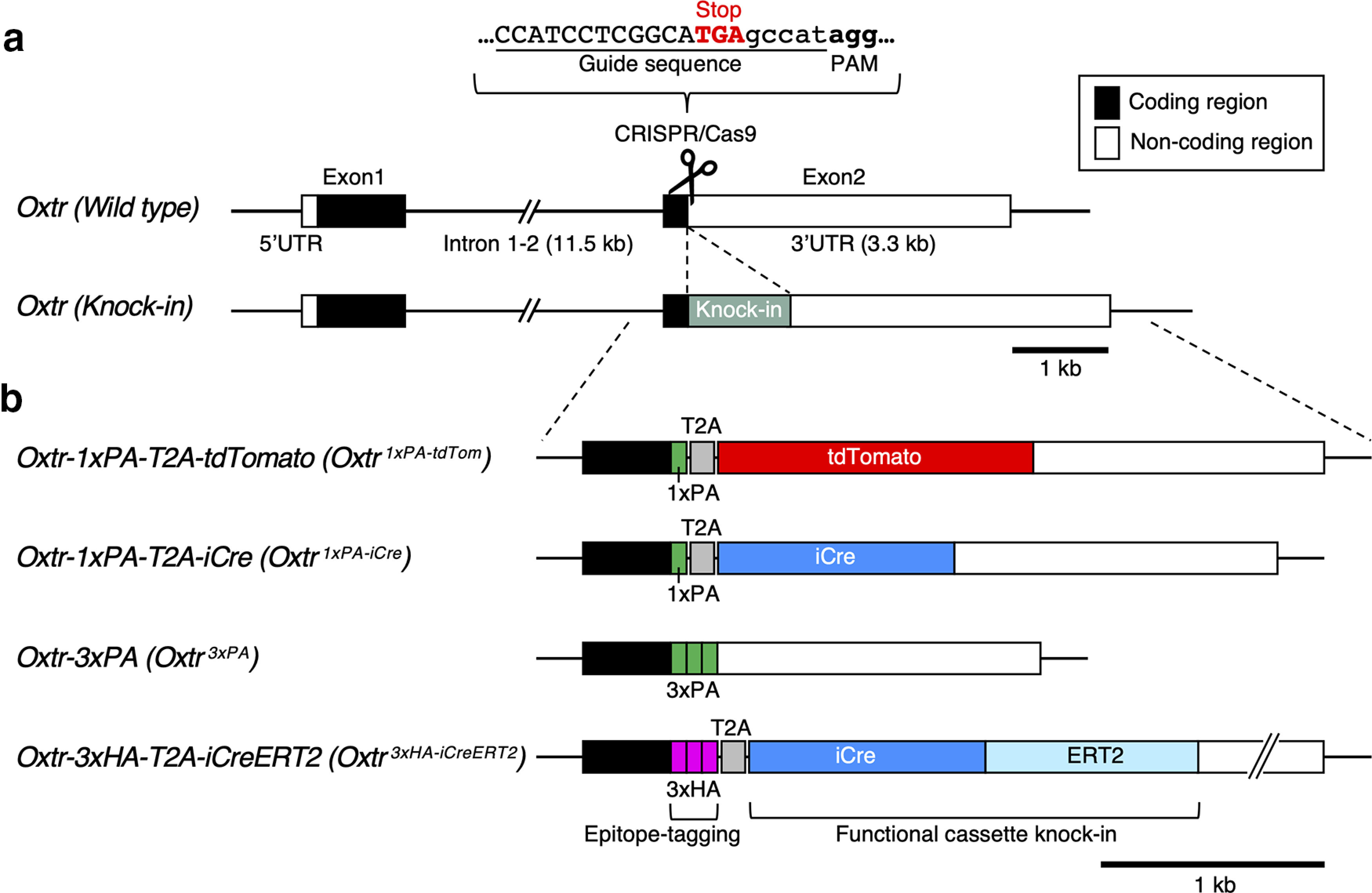
A series of *Oxtr* knock-in mouse lines generated by CRISPR/Cas9 genome editing. ***a***, Targeting strategy to insert a functional cassette just upstream from the translational stop codon (TGA) of mouse *Oxtr* gene locus is schematically outlined. *Oxtr* gene has two coding regions shown as solid black bars that are divided by a long intron. Its 3′ UTR shown as an open bar is also relatively long. PAM and guide sequences to recruit CRISPR components onto the *Oxtr* stop codon are depicted at the top. Solid gray bar indicates a knock-in cassette. Note that the endogenous *Oxtr* gene configuration including the long intron and 3′ UTR are intactly preserved in the resulting knock-in allele even after the genome editing. For comparison with already-existing knock-in lines, see Extended Data Figure 1-1. ***b***, Four knock-in alleles generated in this study are schematically depicted. Epitope tag sequences (1×PA, 3×PA, or 3×HA) are fused 3′ to the Oxtr coding sequences. T2A self-cleaving peptide sequences are employed for bicistronic functional cassette expression (tdTomato, iCre, and ERT2) in Oxtr-expressing cells. Complete genomic DNA sequences and genotyping primer sequences for four knock-in lines are supplied as .dna files (Extended Data 1 DNA sequence files 1-1, 1-2, 1-3, 1-4). Detailed genotyping PCR conditions are listed in Extended Data Figure 1-2.

10.1523/ENEURO.0423-21.2022.f1-1 Extended Data Figure 1-1 Already-existing reporter/Cre lines for Oxtr visualization/manipulation. ***a***, Endogenous *Oxtr* gene configuration is schematically depicted. *Oxtr* gene has two coding regions shown as solid black bars that are divided by a long intron (11.5 kb). Its 3′ UTR shown as an open bar is also relatively long (3.3 kb). ***b***. *Oxtr-Venus* knock-in allele (Yoshida et al., 2009). As the 1st coding exon is replaced with Venus sequences followed by bovine growth hormone (GH) polyA signal, this allele lacks the endogenous configuration of intron and 3′ UTR sequences. ***c***, *Oxtr cDNA^HA^-IRES-Cre* knock-in allele (Hidema et al., 2016). As is the case with *Oxtr-Venus* in ***b***, the 1st coding exon is replaced with *Oxtr cDNA-HA-IRES-Cre* cassette. Although the C-terminally fused HA-tag is detected in uterus tissues, it cannot be detected in brain tissues. ***d***, *Oxtr ^Cre:GFP^* knock-in allele (Ryan et al., 2017). While the long intron is preserved, the exogenously integrated polyA signal is prioritized over the endogenous Oxtr’s polyA. Exogenous polyA signals might affect stabilities of mRNAs. ***e***, *Oxtr-T2A-Cre-D* knock-in allele (Daigle et al., 2018). As is the case in ***d***, the exogenously introduced polyA signal is prioritized over the endogenous Oxtr’s polyA. ***f***, *Oxtr-Cre BAC transgenic* allele (Gerfen et al., 2013). Although bacterial artificial chromosome (BAC) transgenesis is undoubtedly a useful technique, a non-native genomic context via random integration might unexpectedly modify endogenous expression profiles. Download Figure 1-1, TIF file.

Cre recombinase driver mice also play pivotal roles in both conditional knock-out experiments and viral vector-dependent functional analyses. Although two *Oxtr-Cre* knock-in lines using internal ribosome entry site (IRES) sequences for their bicistronic expressions have been generated (Hidema et al., 2016; Ryan et al., 2017; Extended Data Fig. 1-1*c*,*d*), those expression profiles reported were relatively sparse and did not well overlap with each other, probably because of the different context and integrity of their genomic configurations. In addition, the *Oxtr-T2A-Cre-D* line recently became available from The Jackson Laboratory (B6.Cg-Oxtr ^tm1.1(Cre)Hze^/J, stock #031303), generated as a part of project in Allen Institute for Brain Science (Daigle et al., 2018; Extended Data Fig. 1-1*e*), yet its detailed expression profile has not been described.

Endogenously, the Oxtr gene locus consists of two coding exons divided by a long intron (Extended Data Fig. 1-1*a*). In prairie vole and human, some single-nucleotide polymorphisms (SNPs) in this long intron have been reported to be associated with Oxtr expression densities or social behavioral phenotypes (King et al., 2016; Jurek and Neumann, 2018). The authors suggested that these SNPs might overlap with *cis*-regulatory elements in the intron (King et al., 2016). The 3′ UTR of the *Oxtr* gene has also been reported to contain nucleotide variations that are related to social behavioral phenotypes in humans (Jurek and Neumann, 2018). Considering these reports, it must be critical to design knock-in lines by maximally preserving the original transcriptional regulations, so as to precisely recapitulate endogenous Oxtr-expression profiles (Extended Data Fig.1-1 and Gerfen et al., 2013).

For proteins with no antibodies available for immunohistological detection, the epitope tagging strategy could be a promising breakthrough (Brizzard, 2008). Although the previously reported *Oxtr-IRES-Cre* line was designed to harbor an HA-tag at the C terminus of the receptor protein (Extended Data Fig. 1-1*c*), the epitope tag could not be detected in the brain tissues probably because of its relatively lower expression levels, caused by the non-native exon/intron configuration (Hidema et al., 2016). In this study, we first employed the novel PA-tag system in which human podoplanin-derived epitope tag is recognized by its highly specific monoclonal antibody (Fujii et al., 2014). Since this system has been successfully applied to many *in vitro* procedures, such as protein purification and visualization, we anticipated that it would also work *in vivo* to determine the subcellular distributions of endogenous Oxtr protein in neurons. We next used the HA-tag system, because its commercially available antibody (Cell Signaling Technology, C29F4, #3724) has been successfully adopted by *in vivo* genome editing technique, SLENDR, to clearly map the subcellular localization of endogenous proteins in brain tissues with low background staining (Mikuni et al., 2016).

To achieve these concepts, here we apply our refined genome-editing skills to the *Oxtr* locus to generate four novel knock-in mouse lines, and evaluate their expression profiles to ensure the reliability *in vivo*.

## Materials and Methods

### Animals

All animal procedures were performed in accordance with the Animal Ethics Committee’s regulations at the National Institute of Neuroscience. B6C3F1 female mice for fertilized eggs collection and ICR female mice as surrogate mothers were purchased from SLC Japan. One-cell stage zygotes were obtained by mating B6C3F1 stud males with super-ovulated females. Pronuclear injections and mouse transgenesis experiments were performed by standard protocols (Nagy et al., 2003). C57BL/6J mice for back-crossing were purchased from Charles River Japan. B6.Cg-Gt(ROSA)26Sor^tm9(CAG-tdTomato)Hze^/J (also referred to as Ai9, stock #007909) were introduced from The Jackson Laboratory.

### Preparation of CRISPR components

To insert epitope-tag, reporter, and recombinase sequences into the *Oxtr* locus via the CRISPR/Cas9 system, the guide RNA sequences closest to the stop codon TGA in the second exon (depicted in Fig. 1*a*) were selected. Two parts of the CRISPR guide RNA, crRNA and tracrRNA, were chemically synthesized (FASMAC) and recombinant Cas9 protein (EnGen Cas9 NLS S. pyogenes) was purchased from New England Biolabs. For pronuclear injections, equimolar crRNA and tracrRNA were mixed and annealed (94°C, 2 min, then at room temperature for 10 min).

10.1523/ENEURO.0423-21.2022.ed1 Extended Data 1 DNA sequence files (supplied as a zip file). Complete genomic DNA sequences and genotyping primer sequences for four knock-in lines generated in this study are supplied as .dna files that can be opened by using SnapGene software. Download Extended Data 1, ZIP file.

**Figure 2.**
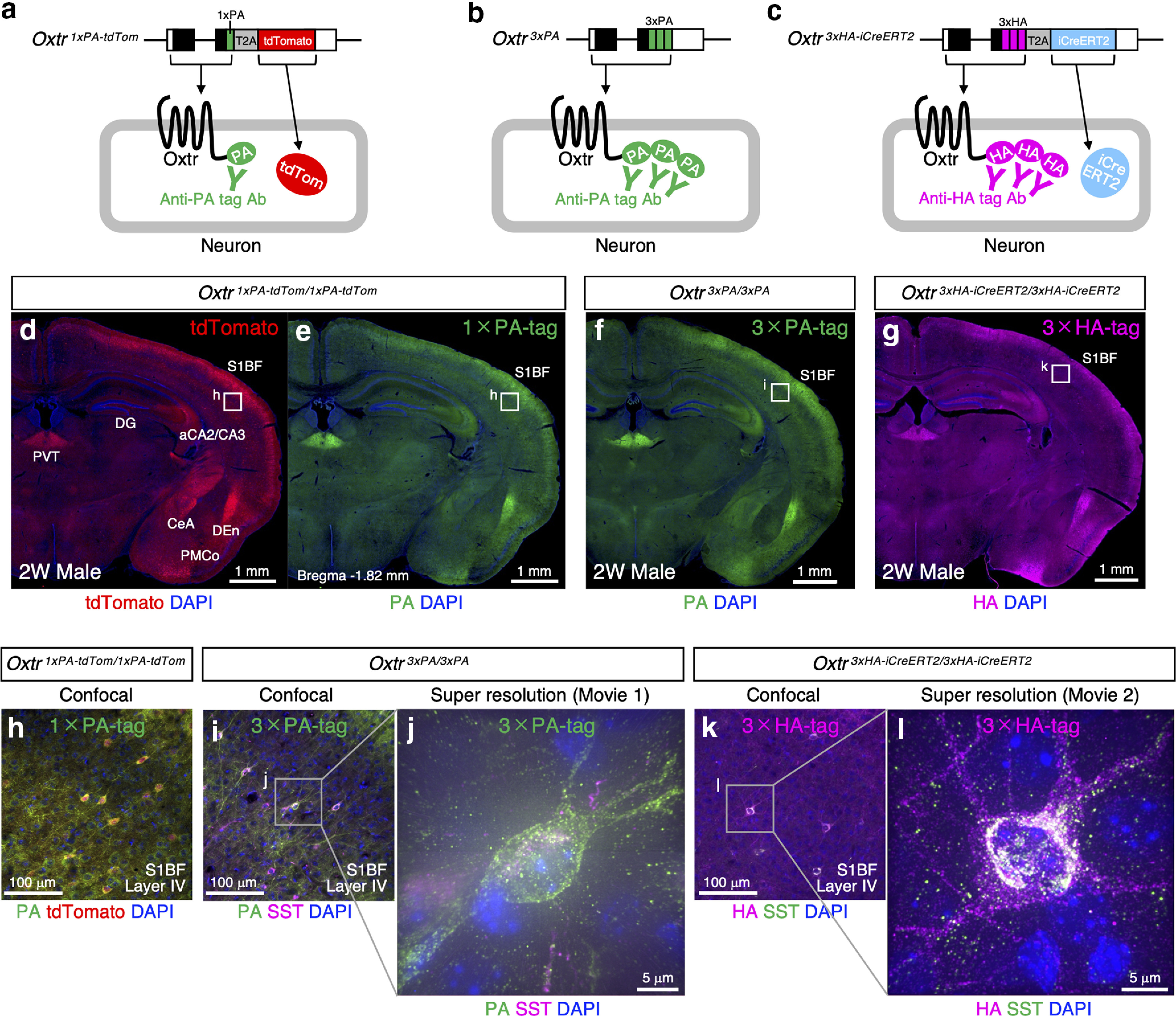
Epitope-tagging strategy precisely visualizes the endogenous Oxtr protein expression profiles and ensures the reliability of knock-in strategies. ***a–c***, Schematic diagram of *Oxtr ^1^*^×^*^PA-tdTom^* allele, *Oxtr ^3^*^×^*^PA^* allele, and *Oxtr ^3^*^×^*^HA-iCreERT2^* allele are arranged on the top. Resulting Oxtr-expressing neurons are depicted below them. To visualize endogenous Oxtr protein localization, PA-tag or HA-tag are fused to the C terminus of receptor protein and detected by anti-tag antibodies. To enhance the detection, three copies of PA-tag or HA-tag are tandemly connected. C-terminally fused PA-tag/HA-tag do not disrupt the receptor function *in vivo* as shown in Extended Data Figure 2-1. tdTomato reporter and iCre-ERT2 recombinase are bicistronically expressed in the cytoplasm by employing the T2A self-cleaving peptide system. ***d***, ***e***, Expression profiles of tdTomato reporter and 1×PA-tag in the *Oxtr ^1^*^×^*^PA-tdTom^* homozygous brain from two-week-old male are arranged (equivalent to bregma −1.82 mm). tdTomato expressions are observed in the areas that have been reported to express Oxtr, such as the S1BF, hippocampal DG and aCA2/CA3, PVT, CeA, DEn, and PMCo. Notably, PA-tag signals are also observed in the same regions, indicating the T2A-dependent bicistronic expression system works well. ***f***, Expression profiles of 3×PA-tag in the *Oxtr ^3^*^×^*^PA^* homozygous brain from two-week-old male nicely match up with those of tdTomato in ***d*** and 1×PA-tag in ***e***. ***g***, Expression profiles of 3×HA-tag in the *Oxtr ^3^*^×^*^HA-iCreERT2^* homozygous brain from two-week-old male well coincide with those of 1xPA-tag in **e** and 3×PA-tag in ***f***. Note that independently generated three epitope-tagged lines result in the same expression profiles, ensuring the reliability of knock-in strategies. ***h***, Magnified confocal image of the boxed area (S1BF, Layer IV) in ***d***, ***e*** indicates tdTomato signals nicely coincide with PA-tag staining. However, this line is not suitable for higher magnification because of its unwanted background staining of PA-tag antibody. ***i***. Enlarged confocal image of the boxed area (S1BF, Layer IV) in ***f*** shows clearer signals derived from 3×PA-tag than those from 1×PA-tag in ***h***. ***j***, Super-resolution image of a SST-positive PA-tagged Oxtr-expressing neuron indicates PA-tag signals are observed both on the surface of cell body and on the neurites, whereas SST is localized in the cytoplasm. 3-D movie of this interneuron is supplied as Movie 1. ***k***, Magnified confocal image of the boxed area (S1BF, Layer IV) in ***g*** shows much clearer signals derived from 3×HA-tag than those from 1×PA-tag in ***h***. ***l***, Super-resolution image of an SST-positive HA-tagged Oxtr-expressing neuron indicates HA-tag signals are observed on the surface of cell body and on the neurites, reconfirming the result from 3xPA-tag knock-in line in ***j***. 3-D movie of this interneuron is supplied as Movie 2.

10.1523/ENEURO.0423-21.2022.f1-2 Extended Data Figure 1-2 Genotyping primers and PCR conditions used in this study. ***a***, Names of four knock-in lines generated in this study are listed. ***b***, Genotyping primer names are listed. ***c***, Primer sequences (5′ to 3′) are listed. ***d***, PCR enzymes used for genotyping are listed. ***e***, PCR products’ size for knock-in alleles and wild-type allele are listed. ***f***, PCR conditions for genotyping are listed. ***g***, Electrophoresis conditions are listed. Download Figure 1-2, TIF file.

### Generation of the *Oxtr ^1^*^×^*^PA-tdTom^* mouse line

To insert 1×PA-tag, T2A self-cleaving peptide, and tdTomato sequences just upstream from the stop codon (Fig. 1*b*), the targeting vector containing those sequences flanked by 2.9-kb homology arms on both sides was constructed by the In-Fusion cloning system (Takara Bio). The mixture of annealed crRNA/tracrRNA, Cas9 protein, and the targeting vector was injected into mouse fertilized eggs at the concentrations of 38, 50, and 25 ng/μl, and the eggs were transferred into the oviduct of surrogate mothers to raise pups.

### Generation of the *Oxtr ^3^*^×^*^PA^* mouse line

To insert three tandem copies of PA-tag sequences just upstream from the stop codon (Fig. 1*b*), 3×PA-tag sequences flanked by 129- and 118-bp homology arms were artificially synthesized (Integrated DNA Technologies). To avoid unwanted excision of PA-tag sequences by the internal recombination among three copies, synonymous substitutions were introduced into each tag sequences. By using this synthesized DNA as the template, long single-stranded DNA (ssDNA) donors were prepared with the Phospho-PCR method (Guide-it Long ssDNA Production System, Takara Bio; Inoue et al., 2021). The mixture of annealed crRNA/tracrRNA, Cas9 protein, and long ssDNA donor was injected into mouse zygotes at the concentrations of 38, 50, and 25 ng/μl, and the zygotes were transferred into the oviduct of surrogate mothers to raise pups.

### Generation of the *Oxtr ^1^*^×^*^PA-iCre^* mouse line

To insert 1×PA-tag sequences, T2A self-cleaving peptide sequences, and codon-improved Cre recombinase (iCre; Shimshek et al., 2002) sequences just upstream from the stop codon (Fig. 1*b*), the targeting vector containing those sequences flanked by 245- and 294-bp homology arms was constructed via the In-Fusion cloning system. By using this vector as the template, long ssDNA donors were synthesized by the Phospho-PCR method. The mixture of annealed crRNA/tracrRNA, Cas9 protein, and long ssDNA donor was injected into mouse zygotes at the concentrations of 38, 50, and 18.5 ng/μl, and the zygotes were transferred into the oviduct of surrogate mothers to raise pups.

### Generation of the *Oxtr ^3^*^×^*^HA-iCreERT2^* mouse line

To insert three copies of HA-tag, T2A self-cleaving peptide, iCre, and mutated ligand binding domain of the human estrogen receptor (ERT2) sequences (Indra et al., 1999) just upstream from the stop codon (Fig. 1*b*), the targeting vector containing those sequences flanked by 2-kb homology arms on both sides was constructed by combining artificial gene synthesis (Integrated DNA Technologies) and In-Fusion cloning system. Synonymous base substitutions were introduced into each HA-tag sequence to obviate unwanted internal recombination among them. The mixture of crRNA/tracrRNA complex, Cas9 protein, and the targeting vector was injected into mouse 1-cell embryos at the concentrations of 50, 100, and 15 ng/μl, and the embryos were transferred into the oviduct of surrogate mothers to raise pups.

### Validation of the founder knock-in mice and their backcrossing

Genomic DNAs were prepared from newborns’ tail by proteinase K treatment in lysis buffer (Takara Bio). Knock-in mice were screened by PCR using Tks Gflex DNA polymerase (Takara Bio) or PrimeSTAR GXL DNA polymerase (Takara Bio). To exclude the possibilities that the donor DNAs had been randomly integrated into the genome, a subset of genotyping primers was designed external to the donor DNAs’ homology arms. For knock-in candidates, PCR products were analyzed by Sanger sequencing to confirm carrying correct insertions. Obtained founders were backcrossed with C57BL/6J to get F1 heterozygous progenies, which were again sequence-verified to harbor the designed knock-in allele. F1 heterozygous mice were then intercrossed to generate homozygous knock-in mice. For histologic, electrophysiological, and viral analyses, homozygous knock-in mice after F3 generations were used.

### Antibodies and viral vectors

Primary antibodies and dilutions used were guinea pig anti-tdTomato (1:2000, Frontier Institute AB_2631185), rat anti-PA-tag (1:2000, FUJIFILM Wako Chemicals NZ-1012-25861), rabbit anti-HA-tag (1:2000, Cell Signaling Technology C29F4 3724S), goat anti-somatostatin (SST; 1:2000, Santa Cruz Biotechnology sc-7819), guinea pig anti-GFP (1:1000, Frontier Institute AB_2571575), rabbit anti-Cux1 (CDP, 1:2000, Santa Cruz Biotechnology sc-13024), and rabbit anti-Oxt (1:2000, IMMUNOSTAR 20068). Appropriate Alexa Fluor-conjugated secondary antibodies were from Thermo Fisher Scientific, Abcam, or Jackson ImmunoResearch and used at dilutions of 1:600.

AAV-PHP.eB CBh_FLEX-GFP WPRE was kindly gifted by Hirokazu Hirai [the program for Brain Mapping by Integrated Neurotechnologies for Disease Studies (Brain/MINDS), AMED].

### Immunohistochemistry

Brain samples were prepared from 2- to 11-week-old male homozygous knock-in mice and immunohistochemically analyzed by standard protocols. Briefly, brains were intracardially perfused with 4% paraformaldehyde (PFA) in PBS (pH 7.4), postfixed with the same fixative for 16 h, and cryo-protected with 30% sucrose in PBS, then embedded in OCT compound (Sakura Finetek Japan) to be frozen. Brains were sliced into 40-μm coronal sections using a Leica cryostat. Sections were treated in blocking buffer (10% normal donkey serum, 0.3% Triton X-100 in PBS) for 1 h at room temperature with gentle agitation. Sections were then incubated with primary antibodies in blocking solution (3% normal donkey serum, 0.3% Triton X-100 in PBS) overnight at 4°C. After being washed with PBS, sections were treated with appropriate Alexa Fluor-conjugated secondary antibodies (Thermo Fisher Scientific, Abcam, or Jackson ImmunoResearch) and DAPI for 1 h at room temperature. After washing, sections were mounted onto glass slides with Permafluor (Thermo Fisher Scientific) or VECTASHIELD Vibrance antifade mounting medium (Vector Laboratories). Fluorescent images were obtained on a KEYENCE BZ-X710. For confocal and super-resolution imaging, an OLYMPUS SpinSR10 was used to take z-stacked images. Movies were constructed by using an OLYMPUS cellSens software.

### Electrophysiological recordings from tdTomato-positive neurons in the *Oxtr ^1^*^×^*^PA-tdTom^* line

Coronal PFC slices (300-μm thickness) were prepared from four- to six-week-old male homozygous *Oxtr ^1^*^×^*^PA-tdTom^* mice, using standard procedures (Miwa et al., 2008). Slices were perfused with artificial CSF (ACSF) that was saturated with 95% O_2_ and 5% CO_2_ at 30 ± 1°C and contained 119 mM NaCl, 3.5 mM KCl, 2.5 mM CaCl_2_, 1.3 mM MgSO_4_, 1.0 mM NaH_2_PO_4_, 26.2 mM NaHCO_3_, and 11 mM D-glucose. Spontaneous spiking in tdTomato-expressing neurons in Layers II and V of the PFC was acquired in voltage clamp cell-attached configuration with a MultiClamp 700B patch-clamp amplifier (Molecular Devices). The pipette solution contained ACSF, and the patch was held at 0 mV. Baseline firing was acquired for 5 min, followed by bath application of 1 μM [Thr^4^, Gly^7^]-Oxt (TGOT) or 1 μM (d(CH_2_)_5_^1^, Tyr(Me)^2^, Thr^4^, Orn^8^, des-Gly-NH_2_^9^)-vasotocin (OTA) for 10 min. The signal was filtered at 1 kHz and digitized at 20 kHz with National Instruments DAQ board (USB-6341) and Igor Pro (Wavemetrics) with the NeuroMatic software package (Rothman and Silver, 2018). TGOT and OTA were obtained from Bachem.

### Intravenous injection of AAV-PHP.eB vector to the *Oxtr ^1^*^×^*^PA-iCre^* line

After anesthesia with isoflurane (4%), 100 μl of viral solution (AAV-PHP.eB CBh_FLEX-GFP WPRE; 2.5 × 10^12^ vg/ml) was intravenously injected into the retro-orbital sinus of *Oxtr ^1^*^×^*^PA-iCre/+^* mice using a 1.0-ml syringe with a 27-gauge needle. Four weeks after the injection, the mice were anesthetized with sodium pentobarbital (50 mg/kg body weight, intraperitoneal) and transcardially perfused with PBS followed by 4% PFA in 0.1 M phosphate buffer (pH 7.4). The brains were removed, postfixed in the same fixative overnight at 4°C, and immersed in 15% and 30% sucrose in PBS overnight for cryoprotection. Free-floating sections (40 μm) were cut using a sliding microtome (REM-710; Yamato Kohki Industrial Co, Ltd.) and incubated in PBS with 0.3% Triton X-100 and 1% bovine serum albumin (BSA) for 1 h at room temperature. Then, the sections were incubated for 12 h at room temperature with primary guinea pig anti-GFP antibody (1:1000; Frontier Institute) diluted in PBS with 0.3% Triton X-100, 2% BSA. The sections were washed in PBS and incubated for 2 h in Alexa Fluor 488-conjugaed species-specific secondary antibodies (Thermo Fisher Scientific) diluted 1:500 in PBS with 0.3% Triton X-100 and 2% BSA. Fluorescent images of GFP were captured using a fluorescent microscope (BZ-X710, KEYENCE).

### Induction of iCre activity in the *Oxtr ^3^*^×^*^HA-iCreERT2^* line by oral administration of TMX

*Oxtr ^3^*^×^*^HA-iCreERT2:^*: *Gt(ROSA)Sor ^Ai9^* double heterozygous males at four weeks were fed with powdered diet supplemented with tamoxifen (TMX; Sigma-Aldrich T5648) at the concentration of 2 mg/g for five consecutive days by using feeding jars (Yoshinobu et al., 2021; *n* = 5). For the control experiments, double heterozygous males at four weeks were fed with normal diet for five consecutive days (*n* = 3). After five more days, brains were sampled at five weeks and immunostained as described above. To analyze effects of chronic TMX treatment on Oxt and Oxtr expressions, five-week-old homozygous *Oxtr ^1^*^×^*^PA-tdTom^* males were fed with the same protocol (*n* = 3). Normal diet was fed to mice with the same genotype as the control (*n* = 3). Brains were sampled at six weeks for immunostaining.

### Material availability

Four knock-in mouse lines generated in this study will be made available with a materials transfer agreement.

## Results

### Seamless insertions of epitope tag and functional gene cassettes into the *Oxtr* locus via CRISPR/Cas9 genome editing

To insert the epitope tag or reporter/recombinase sequences in-frame and 5′ to the stop codon of the *Oxtr* gene second exon, we selected the guide RNA sequences depicted in Figure 1*a*. The targeting strategies were basically the same for four knock-in lines; one or three copies of epitope tag sequences were directly fused 3′ to the Oxtr coding sequences. For bicistronic expression, we inserted T2A self-cleaving peptide sequences followed by functional gene cassettes (tdTomato reporter or Cre recombinase coding sequences). We used the T2A sequences which had been successfully used for knock-in mouse generations, to simultaneously express two separate proteins at nearly equimolar amounts from the same mRNA (Trichas et al., 2008; Tang et al., 2009).

We next employed the cloning-free CRISPR/Cas9 system in which chemically synthesized guide RNAs (crRNA and tracrRNA) were combined with recombinant Cas9 nuclease protein to induce highly efficient genome editing in mouse fertilized eggs (Aida et al., 2015; Inoue et al., 2021), and successfully generated one founder mouse for each knock-in design. We crossed these knock-in founders with C57BL/6J mice to obtain heterozygous F1 generations, which were sequence-verified to ensure that they correctly harbored the designed knock-in cassettes. We then intercrossed F1 heterozygous mice to obtain homozygous F2 generations. For histologic, electrophysiological, and viral analyses, we prepared homozygous knock-in mice after F3 generations. Complete genomic DNA sequences and genotyping primer sequences for the four knock-in lines are supplied as .dna files (Extended Data 1 DNA sequence files). Detailed genotyping PCR conditions are listed in Extended Data Figure 1-2.

### The epitope tagging strategy precisely visualized the endogenous Oxtr protein expression profiles and ensured the reliability of the knock-in strategy

As depicted in Figures 1*b*, 2*a*, we first designed a dual-purpose *Oxtr ^1^*^×^*^PA-tdTom^* allele, in which one copy of PA-tag can monitor endogenous Oxtr protein localization and the portion of the tdTomato cassette serves as a bright fluorescent reporter. To evaluate PA-tag and tdTomato expression profiles in this line, we used homozygous male brains at two weeks [postnatal day (P)14] because Oxtr expression levels in mouse brains have been reported to peak at around two to three weeks (Newmaster et al., 2020). In the figures, we mainly presented the section equivalent to bregma −1.82 mm, as this level contains several well-known regions that have been reported to express Oxtr. Although tdTomato fluorescence could be detected without immunostaining as described later (Extended Data Fig. 3-1*a*,*b*), we double-stained the sections with anti-tdTomato and anti-PA-tag antibodies to validate their colocalization. As a result, we observed abundant tdTomato reporter signals in the cortical region (Newmaster et al., 2020), hippocampal dentate gyrus (DG) and anterior CA2 (aCA2)/CA3 (Raam et al., 2017), paraventricular thalamus (PVT; Newmaster et al., 2020; Watarai et al., 2020), central nucleus of amygdala (CeA; Yoshida et al., 2009; Mitre et al., 2016), dorsal endopiriform nucleus (DEn; Sharma et al., 2019), and posteromedial cortical amygdaloid area (PMCo; Newmaster et al., 2020; Fig. 2*d*), where considerable level of Oxtr expression has already been reported. Consistently, we also detected the presence of endogenous Oxtr protein by the C-terminally fused PA-tag in the same regions (Fig. 2*e*); however, the signal intensity was not strong enough for higher magnification, as described below. These matched results indicated that the epitope tagging and T2A-dependent bicistronic strategies we employed properly functioned as expected. We therefore applied the same design for the *Oxtr ^1^*^×^*^PA-iCre^* allele (Fig. 1*b*).

10.1523/ENEURO.0423-21.2022.f2-1 Extended Data Figure 2-1 Homozygous knock-in males and females do not have any problems in their reproductive functions. ***a***, Pups from *Oxtr* knock-out mothers (*Oxtr ^–/–^*) has been reported to die soon after birth due to mother’s defects in breast-feeding since Oxt plays essential roles in lactation (Hidema et al., 2016). Pups’ digestive tracts are not filled with milk. ***b***, Digestive tract of a pup from homozygous *Oxtr ^1^*^×^*^PA-tdTom^* mother is filled with milk, indicating homozygous mothers have no problems in lactation. ***c***, Digestive tract of a pup from homozygous *Oxtr ^1^*^×^*^PA-iCre^* mother is filled with milk, indicating the peripheral Oxt signaling pathway is intact. ***d***, Mammary glands of homozygous *Oxtr ^1^*^×^*^PA-tdTom^* mother are well-developed. ***e***, A homozygous *Oxtr ^1^*^×^*^PA-iCre^* mother can nurse her pups by lactating. ***f***, A pair of homozygous *Oxtr ^1^*^×^*^PA-iCre^* mother and father, and their homozygous progenies. Homozygous knock-in males are fertile. Download Figure 2-1, TIF file.

In the magnified confocal image of the primary somatosensory cortex barrel field (S1BF) in the *Oxtr ^1^*^×^*^PA-tdTom^* brain (Fig. 2*h*), tdTomato and PA-tag signals showed significant colocalization. However, we noticed that the use of anti-PA-tag antibody resulted in high background staining within brain tissue. To increase the signal-to-noise (S/N) ratio, we next generated the *Oxtr ^3^*^×^*^PA^* allele in which three tandem copies of PA-tags were simply fused to the C terminus of Oxtr (Figs. 1*b*, 2*b*). Consequently, the PA-tag signals from this 3×PA allele (Fig. 2*f*) perfectly coincided with those from 1×PA in the *Oxtr ^1^*^×^*^PA-tdTom^* allele (Fig. 2*e*), and its S/N ratio was improved. On comparing the magnified confocal images of the S1BF, the PA-tag signals in the *Oxtr ^3^*^×^*^PA^* line (Fig. 2*i*) were much clearer than those in the *Oxtr ^1^*^×^*^PA-tdTom^* allele (Fig. 2*h*), along with relatively lower background staining. In Figure 2*i–l*, we focused on SST-expressing interneurons in Layer IV, since Oxtr-expressing interneurons have been found in some brain areas to recruit local inhibitory circuits, which might be a major mode of oxytocinergic modulation in the brain (Froemke and Young, 2021), as described in Discussion.

We further designed a dual-purpose *Oxtr ^3^*^×^*^HA-iCreERT2^* allele (Figs. 1*b*, 2*c*). We newly employed three copies of HA-tags for endogenous Oxtr protein visualization, because a good antibody with lower background staining in the brain tissue was commercially available. To induce spatiotemporally controlled Cre recombinase expressions in mouse brains, we fused a mutated ligand-binding domain of the human estrogen receptor (ERT2; Indra et al., 1999) with iCre recombinase. The detailed results for iCre inductions are described later. As shown in Figure 2*g*, the HA-tag signals from this 3×HA allele nicely coincided with those from the 1×PA and 3×PA alleles (Fig. 2*e*,*f*). On the magnified confocal image of *Oxtr ^3^*^×^*^HA-iCreERT2^* brain shown in Figure 2*k*, we observed clear HA-tag signals, with relatively low background staining. The fact that three independently generated epitope-tagged lines resulted in exactly the same expression profiles confirms the reliability of our knock-in strategies.

### *Oxtr ^3^*^×^*^PA^* and *Oxtr ^3^*^×^*^HA-iCreERT2^* mouse lines enabled visualization of endogenous Oxtr protein at the subcellular level

We next employed spinning-disk confocal microscopy technology to observe epitope tagged Oxtr-expressing neurons at super-resolution. First, we obtained the Z-stacked 3-D image of a 3×PA-tagged neuron in the S1BF, which was identified as an SST-positive interneuron (Fig. 2*j*; Movie 1). We successfully detected the distribution of PA-tagged endogenous Oxtr proteins on the plasma membrane, as expected as a member of GPCRs, whereas SST was localized in the cytoplasm. This is the first evidence showing that Oxtr proteins reside both on the cell body and on nerve fibers. The super-resolution image from a 3×HA-tagged neuron further replicated the above observation, supporting the reliability of this approach (Fig. 2*l*; Movie 2).

Movie 1. Super-resolution image of an SST-positive PA-tagged Oxtr-expressing neuron. PA-tag signals shown in green are observed both on the surface of cell body and on the neurites, whereas SST signals shown in magenta are localized in the cytoplasm. DAPI nuclear staining signals are shown in blue. 10.1523/ENEURO.0423-21.2022.video.1

Movie 2. Super-resolution image of an SST-positive HA-tagged Oxtr-expressing neuron. HA-tag signals shown in magenta are observed both on the surface of cell body and on the neurites, whereas SST signals shown in green are localized in the cytoplasm. DAPI nuclear staining signals are shown in blue. 10.1523/ENEURO.0423-21.2022.video.2

### C-terminally fused epitope tags did not disrupt the Oxtr protein function *in vivo*

**Figure 3.**
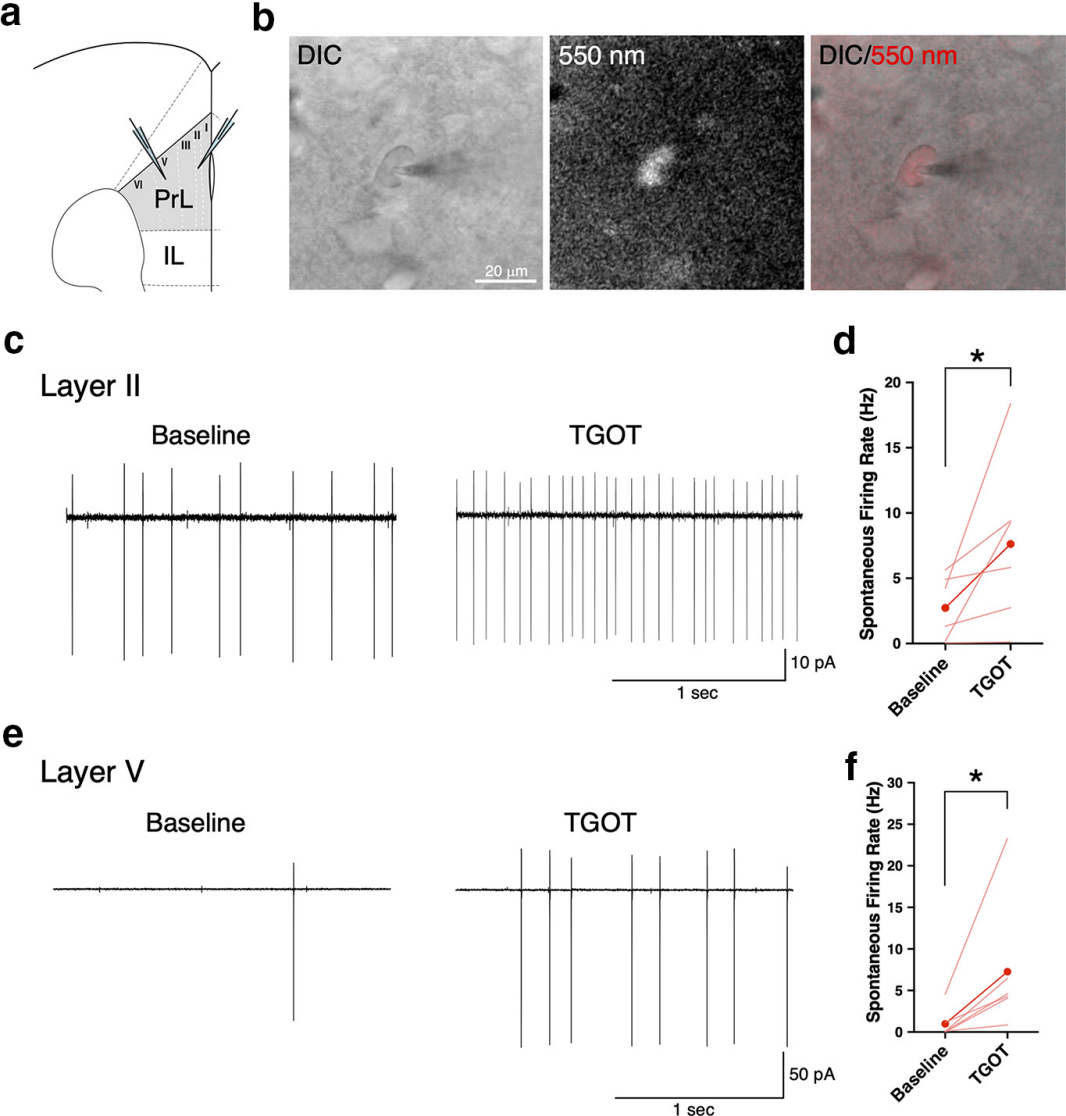
Functional verification of tdTomato-positive neurons in *Oxtr ^1^*^×^*^PA-tdTom^* mice by *in vitro* electrophysiological analyses. ***a***, Schematic diagram of a mouse brain coronal section which shows placement of recording electrodes in Layer II and Layer V of the PrL. IL, infralimbic cortex. Distribution of tdTomato-positive neurons in the PrL of *Oxtr ^1^*^×^*^PA-tdTom^* homozygous brain are shown in Extended Data Figure 3-1. ***b***, Representative differential interference contrast (DIC; left), fluorescent (red; middle), and merged (right) images of a Layer V neuron in the PrL from four-week-old *Oxtr ^1^*^×^*^PA-tdTom^* homozygous male are arranged. ***c***, Representative cell-attached recording traces from a Layer II tdTomato-positive cell in the PrL. Left panel shows the baseline firing rate. Upon treatment of Oxtr agonist, TGOT, the firing rates are significantly increased as shown in right panel. Scale bar: 10 pA, 1 s. ***d***, Quantification of spontaneous firing activities from Layer II tdTomato-positive cells in the PrL (*n *=* *6 cells from 6 mice). Light red lines represent individual neurons’ responses. Red circles with line represent average response. Presence of 1 μM TGOT significantly increases the firing rate; *p <* *0.05 by Wilcoxon’s test. As shown in Extended Data Figure 3-2*a*, this TGOT-induced increase in firing rates is inhibited by presence of Oxtr antagonist, OTA. ***e***, Representative cell-attached recording traces from a Layer V tdTomato-positive cell in the PrL. Left panel indicates the baseline firing rate. On treatment of TGOT, the firing rates are considerably increased as shown in right panel. Scale bar: 50 pA, 1 s. ***d***, Quantification of firing activities from Layer V tdTomato-positive cells in the PrL (*n *=* *6 cells from 6 mice). Administration of 1 μM TGOT considerably increases the spontaneous firing rate; *p <* *0.05 by Wilcoxon’s test. As shown in Extended Data Figure 3-2*b*, this TGOT-induced increase in firing rates is inhibited by presence of OTA.

Although our experiments have confirmed the advantage of epitope tagging in visualizing endogenous protein localization, the concern remained that C-terminally fused tags may alter Oxtr protein conformation and/or stability, affecting its functions. Since Oxt plays essential roles in lactation, pups from *Oxtr* knock-out mothers (*Oxtr* ^−/−^) have been reported to die soon after birth, because of the mother’s defects in breastfeeding (Takayanagi et al., 2005; Hidema et al., 2016; Extended Data Fig. 2-1*a*). Therefore, we confirmed that all the homozygous mothers of our four knock-in lines had successfully nursed their pups. As shown in Extended Data Figure 2-1, the pups’ digestive tracts were filled with milk (Extended Data Fig. 2-1*b*,*c*) and the mother’s mammary glands were well developed (Extended Data Fig. 2-1*d*), indicating that the peripheral Oxt signaling pathway was intact in the homozygous females. In addition, we performed electrophysiological recordings in tdTomato-positive neurons from the homozygous *Oxtr ^1^*^×^*^PA-tdTom^* male, to confirm their responsiveness to agonist administration (details are shown in Fig. 3 and described later), supporting that 1×PA tagged Oxtr proteins could sufficiently mediate Oxt signals in the brain. These results indicate that fusion of one or three copies of the PA-tag or HA-tag to the C terminus of Oxtr did not affect receptor functions *in vivo*. It has already been reported that a much larger tdTomato protein fused C-terminally to the κ opioid receptor, which is also a member of the GPCR family, did not disrupt neuronal circuitries in the knock-in mouse brain (Chen et al., 2020). Therefore, we did not expect that the relatively smaller epitope tags we had used in the present study would affect Oxtr functions. Consistent with this assumption, all the knock-in lines we generated could be maintained by crossing homozygous males with homozygous females (Extended Data Fig. 2-1*e*,*f*), without the need for laborious genotyping procedures. This is a notable advantage of our new lines.

10.1523/ENEURO.0423-21.2022.f3-1 Extended Data Figure 3-1 Characteristics of tdTomato-positive neurons in the PrL. Serial coronal sections of five-week-old homozygous *Oxtr ^1^*^×^*^PA-tdTom^* male brain at bregma 1.94 mm are arranged. ***a***, Endogenous tdTomato fluorescence can be detected without immunostaining. ***b***, Enlarged image of the boxed area in ***a***. ***c***, Enhanced tdTomato fluorescent signals are detected with anti-tdTomato antibody. ***d***, Enlarged image of the boxed area in **c** indicates tdTomato-positive cells reside in both Layer II/III and in Layer V of the PrL. ***e***, ***f***, tdTomato-expressing neurons in Layer II express Cux1, one of the glutamatergic neuron markers. ***g***, ***h***, tdTomato-expressing neurons in Layer V express SST, one of the interneuron markers. Download Figure 3-1, TIF file.

10.1523/ENEURO.0423-21.2022.f3-2 Extended Data Figure 3-2 Blocking effects of Oxtr antagonist, OTA on TGOT-induced increase of spontaneous firing activities in tdTomato-positive neurons. ***a***, Mean spontaneous firing activities of Layer II tdTomato-positive cells in the PrL from *Oxtr ^1^*^×^*^PA-tdTom^* homozygous males. Light red lines represent individual neurons’ responses. Red circles with line represent average response. Firing activities remain unchanged in the presence of 1 μm OTA (left panel, *n *=* *6 cells from 4 mice); 1 μm TGOT application in the presence of 1 μm OTA does not increase firing activities of tdTomato-positive cells (right panel, *n *=* *3 cells from 3 mice). ns, not significant. ***b***, Mean spontaneous firing activities of Layer V tdTomato-positive cells in the PrL from *Oxtr ^1^*^×^*^PA-tdTom^* homozygous males are arranged. Firing activities remain unchanged the in presence of 1 μM OTA (left panel, *n *=* *7 cells from 6 mice); 1 μm TGOT application in the presence of 1 μm OTA does not affect firing rates of tdTomato-positive cells (right panel, *n *=* *5 cells from 3 mice). Download Figure 3-2, TIF file.

### The tdTomato reporter in the *Oxtr ^1^*^×^*^PA-tdTom^* line precisely labeled Oxtr-expressing neurons

Since tdTomato has been developed as a nonaggregating, extremely bright red fluorescent protein suitable for live imaging (Shaner et al., 2004), we were able to detect tdTomato signals in our homozygous *Oxtr ^1^*^×^*^PA-tdTom^* brains without immunostaining (Extended Data Fig. 3-1*a*,*b*), allowing us to perform patch-clamp recordings on these cells (Fig. 3*a*,*b*). We hypothesized that tdTomato-positive neurons should correctly respond to an Oxtr agonist, TGOT, if the tdTomato expressions were faithfully regulated parallel to endogenous Oxtr expressions.

A subset of both glutamatergic and GABAergic neurons in the PFC has been reported to express Oxtr, which modulate female sociosexual behavior (Nakajima et al., 2014) and social memory (Tan et al., 2019). We first investigated the distribution of tdTomato-positive neurons in the PFC using immunohistochemistry, particularly in the prelimbic cortex (PrL) of *Oxtr ^1^*^×^*^PA-tdTom^* homozygous brain (Extended Data Fig. 3-1). We found that tdTomato-positive neurons mainly resided in Layers II and V of the PrL (Extended Data Fig. 3-1*c*,*d*). Consistent with a previous report (Tan et al., 2019), we identified the neurons in Layer II by Cux1 expression as glutamatergic neurons (Extended Data Fig. 3-1*e*,*f*), while the neurons in Layer V were confirmed by SST expression as GABAergic interneurons (Extended Data Fig. 3-1*g*,*h*).

To test whether these tdTomato-positive neurons in *Oxtr ^1^*^×^*^PA-tdTom^* mice faithfully respond to TGOT application, we used four- to six-week-old homozygous *Oxtr ^1^*^×^*^PA-tdTom^* males and perform *in vitro* electrophysiological analyses using a cell-attached patch-clamp configuration (Fig. 3*a*,*b*). We tested the neurons in Layers II and V separately (Fig. 3*a*), to test the possibility that they might have distinct responsiveness to Oxt ligands to modulate synaptic sites or membrane potentials. As the results, in tdTomato-positive Layer II neurons, TGOT significantly increased the spontaneous firing rate from 2.7 ± 1.0 to 7.6 ± 2.6 Hz (*n* =* *6; *p *=* *0.0312, Wilcoxon test; Fig. 3*c*,*d*). In tdTomato-positive Layer V neurons, TGOT also considerably increased the spontaneous firing rate from 0.99 ± 0.7 to 7.3 ± 3.3 Hz (*n *=* *6; *p *=* *0.0312, Wilcoxon test; Fig. 3*e*,*f*). Additionally, we confirmed that these TGOT-induced increases in spontaneous firing rates in both Layer II and V neurons were inhibited by the presence of an Oxtr antagonist, OTA (Extended Data Fig. 3-2*a*,*b*). These results clearly demonstrate that tdTomato-positive cells in *Oxtr ^1^*^×^*^PA-tdTom^* mice express “functional” Oxtr and appropriately respond to its ligand, even in the presence of a C-terminally fused PA-tag. Hence, tdTomato reporter signals in our *Oxtr ^1^*^×^*^PA-tdTom^* line are a reliable marker for Oxtr-expressing cells.

### The *Oxtr ^1^*^×^*^PA-iCre^* line sufficiently drove recombinase activities in the Oxtr-expressing neurons

**Figure 4.**
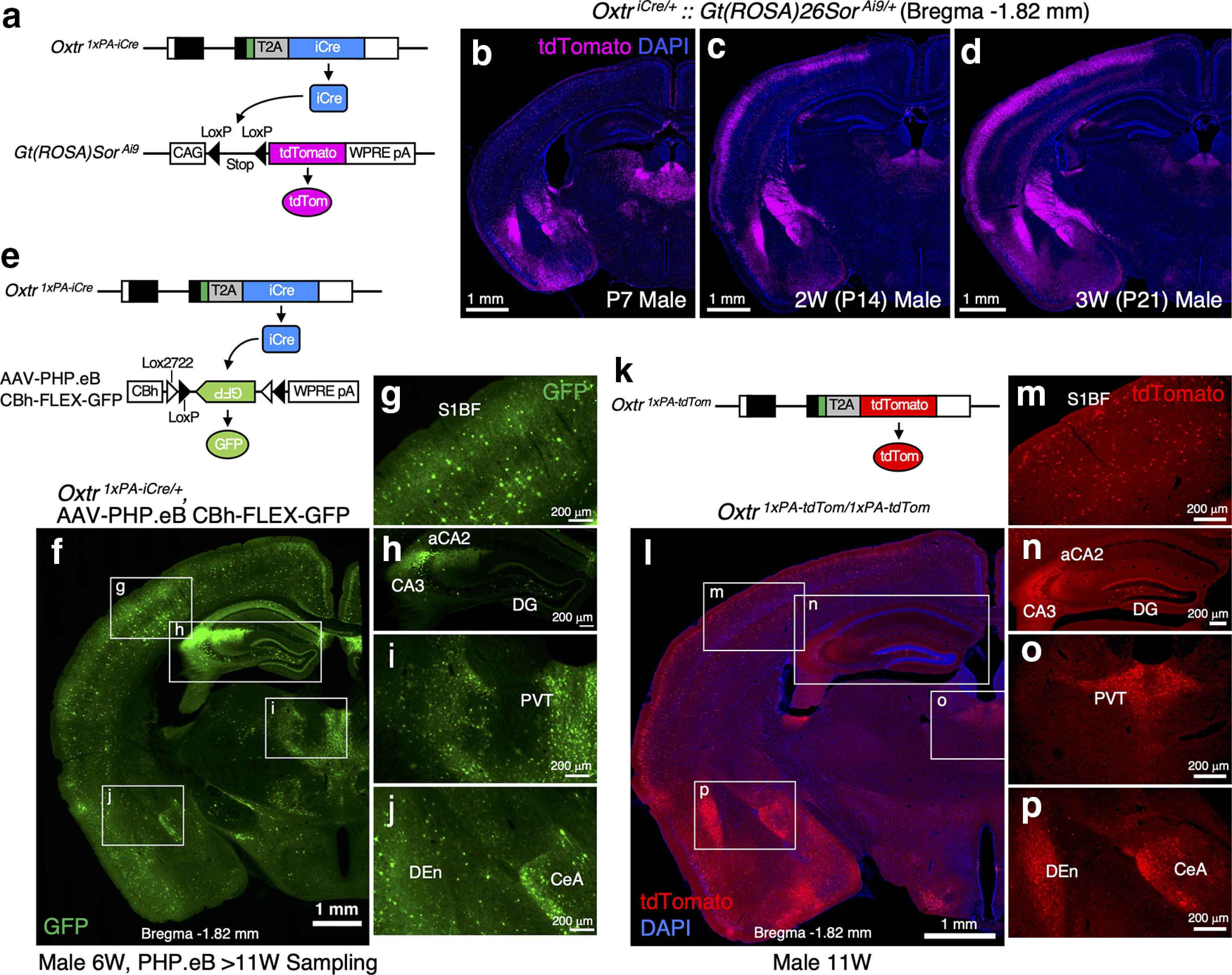
*Oxtr ^1^*^×^*^PA-iCre^* mice reliably drive recombinase activities in Oxtr-expressing cells. ***a***, Schematics of *Oxtr ^1^*^×^*^PA-iCre^* allele and *Gt(ROSA)Sor ^Ai9^* allele are depicted. ***b–d***, tdTomato expression profiles in *Oxtr ^1^*^×^*^PA-iCre/+^*::*Gt(ROSA)Sor ^Ai9/+^* male brains at P7, P14, and P21 are arranged (equivalent to bregma −1.82 mm). Constitutive tdTomato expressions from the *Gt(ROSA)Sor* locus after Cre-dependent stop signal excision gradually accumulate over time. tdTomato signals are observed already at P7 and then become more abundant at P14 and P21. ***e***, Schematics of *Oxtr ^1^*^×^*^PA-iCre^* allele and AAV-PHP.eB vector harboring CBh_FLEX-GFP. GFP expression can be induced by Cre-dependent inversion. ***f***, Expression profiles of GFP in the *Oxtr ^1^*^×^*^PA-iCre^* heterozygous brain (bregma −1.82 mm) from 11-week-old male to which AAV-PHP.eB vector has been retro-rbitally injected at six weeks. Other section levels are shown in Extended Data Figure 4-1. ***g–j***, Enlarged images of the boxed areas in ***f*** are arranged. GFP expressions are observed in the areas that have been reported to express Oxtr, such as the S1BF, hippocampal DG and aCA2/CA3, PVT, CeA, and DEn, indicating this mouse line sufficiently drives Cre activities in Oxtr-expressing cells. ***k***, Schematic diagram of *Oxtr ^1^*^×^*^PA-tdTom^* allele is depicted. ***l***, tdTomato expression profiles in the *Oxtr ^1^*^×^*^PA-tdTom^* homozygous brain (bregma −1.82 mm) from 11-week-old male are arranged. Other section levels are shown in Extended Data Figure 4-1. ***m–p***, Enlarged images of the boxed areas in ***l*** are arranged. These tdTomato expression profiles correspond to the GFP expressions shown in ***g–j*** induced by iCre on AAV-PHPeB administration.

To newly generate an effective Cre driver, we employed the 2A peptide-based bicistronic expression system (Trichas et al., 2008) and codon-improved Cre recombinase (iCre) sequences (Shimshek et al., 2002). For the evaluation of Cre activities in the brain, we first crossed our *Oxtr ^1^*^×^*^PA-iCre^* line with the well-known Cre reporter, Ai9 (Madisen et al., 2010) to obtain double heterozygous mice. As depicted in Figure 4*a*, Cre-dependent stop signal excision induces tdTomato expression from the Ai9 allele. We analyzed tdTomato expression profiles in *Oxtr ^1^*^×^*^PA-iCre/+^::Gt(ROSA)Sor ^Ai9/+^* male brains at P7, P14, and P21. We observed tdTomato signals already at P7 (Fig. 4*b*) and found that the reporter signals became more abundant at P14 and P21 (Fig. 4*c*,*d*). Once recombination occurs, the reporter gene remains recombined thereafter, and consequently, the reporter is expressed even in the absence of ongoing recombinase expressions. In other words, a significant proportion of tdTomato expression in adult stages does not reflect real-time Cre activity but instead reflects a cumulative effect of earlier events. Therefore, we concluded that the Ai9 reporter was unsuitable for our purpose of evaluating Cre expressions in adult brains.

10.1523/ENEURO.0423-21.2022.f4-1 Extended Data Figure 4-1 Atlas of iCre/tdTomato expression profiles. Left side, Expression profiles of GFP in *Oxtr ^1^*^×^*^PA-iCre^* heterozygous brain from 11-week-old male to which AAV-PHP.eB CBh_FLEX-GFP vector has been retro-orbitally injected at six weeks. Right side, tdTomato expression profiles in *Oxtr ^1^*^×^*^PA-tdTom^* homozygous brain from 11-week-old male. ***a***, ***b***, Bregma 1.78 mm. M1, primary motor cortex; S1, primary somatosensory cortex; DEn, dorsal endopiriform claustrum; IEn, intermediate endopiriform claustrum. ***c***, ***d***, Bregma 1.42 mm. Cg, cingulate cortex; S1, primary somatosensory cortex; AcbC, accumbens nucleus, core; AcbSh, accumbens nucleus, shell. ***e***, ***f***, Bregma 0.62 mm. ***g***, ***h***, Bregma −0.10 mm. LS, lateral septum. ***i***, ***j***, Bregma −0.58 mm. ***k***, ***l***, Bregma −2.30 mm. V2M, secondary visual cortex medial area; V1, primary visual cortex; V2L, secondary visual cortex lateral area; Au, auditory cortex. ***m***, ***n***, Bregma −2.92 mm. Download Figure 4-1, TIF file.

We next tried to immunostain Cre proteins in the brain tissue, using a commercially available anti-Cre recombinase antibody (clone 2D8, MAB3120, MERCK). This antibody has been reported to be successful in staining Cre proteins in transgenic lines carrying multiple copies of transgene (Mizuguchi et al., 2012). However, we could not detect Cre in the brain tissues of our iCre knock-in mouse line using this antibody.

To circumvent these issues, we employed AAV-PHP.eB vector harboring CBh-FLEX-GFP (kindly gifted by Hirokazu Hirai, Brain/MINDS project). AAV-PHP.eB has been developed to achieve efficient transport across the blood-brain barrier in adult mammalian brains for broad gene delivery (Chan et al., 2017). As depicted in Figure 4*e*, GFP expressions can be induced by Cre-dependent inversion. We therefore retro-orbitally injected AAV-PHP.eB vector into six-week-old heterozygous males and analyzed their brains after four weeks. As the results, we observed abundant GFP signals in the cortical region, hippocampal DG and aCA2/CA3, PVT, CeA, DEn, and so forth, where Oxtr expression has previously been reported (Fig. 4*f–j*, bregma −1.82 mm). Other section levels are shown in Extended Data Figure 4-1.

As we had designed the *Oxtr ^1^*^×^*^PA-tdTom^* and *Oxtr ^1^*^×^*^PA-iCre^* alleles based on the same concepts (Figs. 1*b*, 4*e*,*k*), their expression profiles should closely resemble each other. Predictably, we found that the tdTomato expression profiles from 11-week-old homozygous *Oxtr ^1^*^×^*^PA-tdTom^* male brains (Fig. 4*l–p*) were quite similar to those of iCre visualized by AAV-PHP.eB administration (Fig. 4*f–j*). Other section levels, shown in Extended Data Figure 4-1, confirm the trend. These results ensured the reliability of our new Cre driver line.

### A new genetic tool, *Oxtr ^3^*^×^*^HA-iCreERT2^* line, could be used to target Oxtr-expressing neurons in a spatiotemporally controlled manner

**Figure 5.**
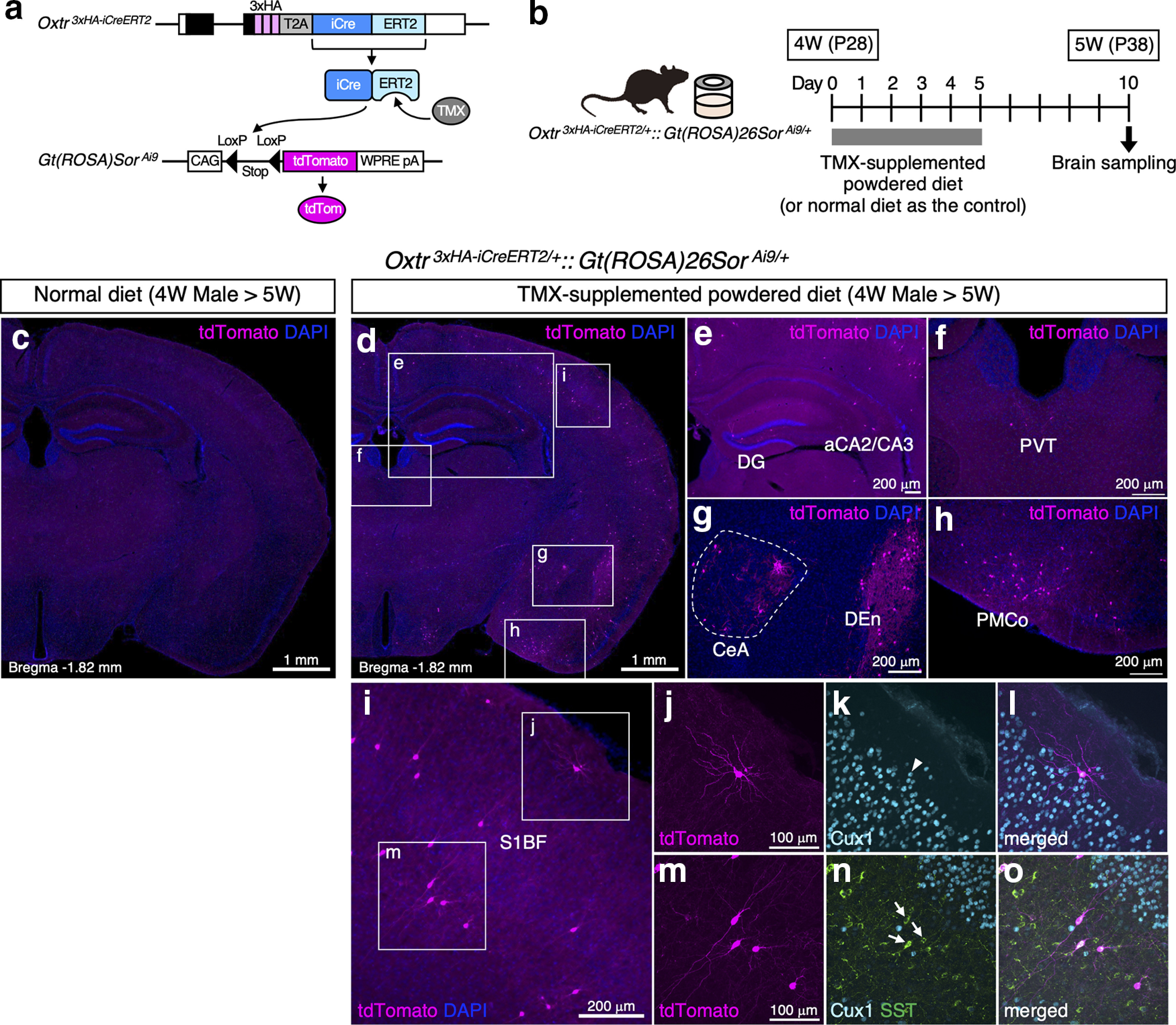
Cre recombinase activities can be reliably induced in Oxtr-expressing neurons on TMX administrations to the *Oxtr ^3^*^×^*^HA-iCreERT2^* line. ***a***, Schematics of *Oxtr ^3^*^×^*^HA-iCreERT2^* allele and *Gt(ROSA)Sor ^Ai9^* allele are depicted. TMX binds to iCre-ERT2 expressed in the cytoplasm, resulting in iCre translocation into the nucleus to flip out stop signal from Ai9 allele and induce tdTomato expression. ***b***, Schematic diagram of experimental protocol. *Oxtr ^3^*^×^*^HA-iCreERT2^*::*Gt(ROSA)Sor ^Ai9^* double heterozygous mice can freely access to TMX-supplemented diet from day 0 to day 5. At day 10, brains are sampled and immunostained. For control experiments, TMX-free normal diet is fed to same genotype mice. As shown in Extended Data Figure 5-1, 5-d TMX treatment has no effects on Oxt/Oxtr expression profiles in five-week-old males. ***c***, TMX-free normal diet does not induce any leaky tdTomato signals in the brain from five-week-old male (bregma −1.82 mm). ***d***, TMX-supplemented diet induces tdTomato expressions in the cortical region, hippocampal DG and aCA2/CA3, PVT, CeA, DEn, and PMCo, where Oxtr expressions has been reported. The density of the tdTomato-positive cells is much sparser than that induced by the constitutive iCre line shown in Figure 4*d*. ***e–h***, Enlarged images of the boxed areas in ***d*** are arranged. ***i***, Enlarged image of the S1BF in ***d***. ***j–l***, Confocal image of the boxed area in ***i***, labeled with Cux1 immunostaining, reveals the morphology of Oxtr-expressing glutamatergic neuron in Layer II. White arrowhead in ***k*** indicates the colocalization of Cux1 and tdTomato. ***m–o***, Confocal images of the boxed area in ***i***, labeled with Cux1 and SST immunostaining, reveals the morphologies of Oxtr-expressing SST-positive interneurons in Layer IV. White arrows in ***n*** indicate the colocalization of SST and tdTomato.

We next investigated whether the *Oxtr ^3^*^×^*^HA-iCreERT2^* allele properly functioned as an inducible Cre driver. To detect Cre activity in the brain, we crossed the *Oxtr ^3^*^×^*^HA-iCreERT2^* line with Ai9 to obtain double heterozygous mice. As depicted in Figure 5*a*, TMX binds to iCre-ERT2 expressed in the cytoplasm, resulting in iCre translocation into the nucleus to flip out the stop signal from Ai9 allele and elicit tdTomato expressions. We orally administered TMX to four-week-old males for five consecutive days by feeding them a TMX-supplemented powdered diet (Fig. 5*b*). For the control experiments, we fed four-week-old males with normal diet for five consecutive days. After five more days, we sampled and analyzed their brains at the level of bregma −1.82 mm. As shown in Figure 5*c*, the normal diet did not induce any leaky tdTomato reporter expression. Contrarily, the TMX-containing diet successfully induced iCre recombinase activity in the cortical region, hippocampal DG and aCA2/CA3, PVT, CeA, DEn, and PMCo, where Oxtr expression has previously been reported (Fig. 5*d–i*). In the constitutive iCre line, too many cells were labeled with tdTomato to be able to clearly distinguish each neuron (Fig. 4*d*). However, reporter expression was only sparsely induced in the inducible iCre line, allowing the identification of cell morphology at single-cell resolution (Fig. 5*d–i*). As a result, we observed two types of Oxtr-expressing neurons with different morphologies and properties in the S1BF (Fig. 5*i*). Consistent with the previous reports (Nakajima et al., 2014; Tan et al., 2019), some tdTomato-labeled neurons in Layer II turned out to be Cux1-positive glutamatergic neurons (Fig. 5*j–l*), while other tdTomato-labeled neurons in Layer IV were identified as SST-positive interneurons (Fig. 5*m–o*).

10.1523/ENEURO.0423-21.2022.f5-1 Extended Data Figure 5-1 Five-day TMX treatment has no effect on Oxt/Oxtr expression profiles in male *Oxtr ^1^*^×^*^PA-tdTom/1^*^×^*^PA-tdTom^* mice. ***a***, Schematic diagram of experimental protocol. Five-week-old homozygous *Oxtr ^1^*^×^*^PA-tdTom^* males can freely access to TMX-supplemented diet from day 0 to day 5 (*n* = 3). At day 10, brains are sampled for immunostaining. For control experiments, TMX-free normal diet is fed to mice with the same genotype (*n* = 3). ***b***, Oxt expression profiles in respective *Oxtr ^1^*^×^*^PA-tdTom/1^*^×^*^PA-tdTom^* male brain are arranged (bregma −0.94 mm). Left column shows Oxt-expressing neurons in the PVN and SON from normal diet conditions. In right column, 5-d TMX treatment has no effect on Oxt expression profiles in these nuclei. ***c***, tdTomato expression profiles in respective *Oxtr ^1^*^×^*^PA-tdTom/1^*^×^*^PA-tdTom^* male brain are arranged (bregma 0.62 mm). In this knock-in line, tdTomato monitors real-time Oxtr expressions. Left column shows tdTomato-expressing cells in the BNST from normal diet conditions. In right column, 5-d TMX treatment has no effect on tdTomato expressions in the BNST. ***d***, tdTomato expression profiles in respective *Oxtr ^1^*^×^*^PA-tdTom/1^*^×^*^PA-tdTom^* male brain are arranged (bregma −1.82 mm). Left column shows tdTomato-expressing cells in the VMH, MeA, PMCo, and DEn from normal diet conditions. In right column, 5-d TMX treatment has no effect on tdTomato expressions in these brain areas. Download Figure 5-1, TIF file.

These results demonstrated that the first-time inducible-Cre driver for Oxtr-expressing neurons efficiently drives Cre-mediated recombination in a spatiotemporally controlled manner on TMX administration.

Since subcutaneous implantations of a pellet containing estrogen into gonadectomized rodents have been reported to affect Oxt/Oxtr transcript levels (Bale and Dorsa, 1995; Vanya et al., 1997; Nomura et al., 2002; Patisaul et al., 2003; Garcia et al., 2017) and radioactively labeled OTA binding to Oxtr (Young et al., 1998), chronic administrations of TMX, a selective estrogen receptor modulator, might change Oxt/Oxtr expression levels in the *Oxtr ^3^*^×^*^HA-iCreERT2^* line. To analyze the effect of 5-d oral TMX treatment on Oxt/Oxtr expressions, we used homozygous *Oxtr ^1^*^×^*^PA-tdTom^* mice, in which real-time Oxtr expressions can be monitored as tdTomato signals, and examined Oxt-expressing neurons by immunostaining (Extended Data Fig. 5-1*a*). Although long-term (12 d to three months) estrogen treatments have been reported to significantly increase Oxt transcript levels in the paraventricular hypothalamic nucleus (PVN; Nomura et al., 2002; Patisaul et al., 2003) and supraoptic nucleus (SON; Garcia et al., 2017), our relatively short-term (5 d) TMX treatment did not affect Oxt expression levels in these nuclei (Extended Data Fig. 5-1*b*). In addition, while long-term estrogen treatments have been reported to affect Oxtr transcript levels in the bed nucleus of stria terminalis (BNST; Garcia et al., 2017), ventromedial hypothalamus (VMH; Bale and Dorsa, 1995; Vanya et al., 1997), and medial amygdala (MeA; Garcia et al., 2017), our 5-d TMX treatment had no effect on Oxtr expression profiles in these brain areas (Extended Data Fig. 5-1*c*,*d*). Furthermore, while radioactively labeled OTA binding to Oxtr has been reported to increase in the DEn and PMCo by long-term estrogen treatment (Young et al., 1998), Oxtr expression levels itself were not affected on our TMX administrations (Extended Data Fig. 5-1*d*). From these results, we concluded that 5-d TMX treatments have little effect on Oxt/Oxtr expression profiles in young male mice. However, we need to carefully interpret the results obtained from *Oxtr ^3^*^×^*^HA-iCreERT2^* line by chronic TMX administrations because estrogens play key roles in mediating social behaviors (Laredo et al., 2014; Ervin et al., 2015) as described in Discussion.

## Discussion

Genetic tools that provide access to Oxtr-expressing cells remain limited. To improve this situation, we have newly generated a series of genome-edited mouse lines that are to make a valuable contribution to the growing research on Oxt. We have created simple and seamless knock-in designs that maximally preserve the endogenous transcriptional regulation of the *Oxtr* gene.

The first achievement of the present study was the successful use of epitope tags to visualize Oxtr protein distributions at the subcellular level for the first time. Two strategies, the RNAscope-based *in situ* hybridization and the *Oxtr-Venus* knock-in mice, have previously been used for Oxtr visualization. However, these methods detect transcript expression, but not Oxtr protein localization per se. To overcome these limitations, we generated epitope tag knock-in mice by genome editing. We employed the PA-tag, a 12-aa epitope derived from human podoplanin, which could be detected using the rat monoclonal antibody, NZ-1 (Fujii et al., 2014). This epitope-antibody combination has been reported to show a high binding affinity of K_D_ = 4.9 × 10^−10^ (M), which is much stronger than those of HA, FLAG, and other affinity tag systems, and which has been successfully used to many biological procedures *in vitro* (Fujii et al., 2014). In addition, we have previously reported the applicability of the PA-tag system in mouse brains and testes in *in vivo* studies (Arimura et al., 2020; Kimura et al., 2021). Here, we triplicated the PA-tag to improve the S/N ratio of immunostaining, by which the background staining in brain tissues were reduced, allowing us to successfully carry out super-resolution imaging of Oxtr protein. We also used the HA-tag because it has been successfully employed for SLENDR, which enabled *in vivo* protein labeling via genome editing in mammalian brains (Mikuni et al., 2016). Similarly, using three copies of the HA-tag enabled us to reconfirm the result from the 3×PA tag knock-in brains.

Although we have roughly demonstrated that Oxtr proteins are present not only on cell bodies but also on neurites, it remains unclear on which part of neurites (axons, dendrites, or both) they reside. To date, three types of oxytocinergic modulation on neurons have been proposed (Froemke and Young, 2021). As the first-order modulation, Oxt directly depolarizes excitatory cells that express Oxtr. In the second-order modulation, Oxt acts on inhibitory interneurons expressing Oxtr, to regulate synaptic inhibitions. Furthermore, in the third-order modulation, Oxtr is believed to reside on axon terminals, where the presynaptic Oxtr activation enhances transmitter release and indirectly impacts on other excitatory transmissions (Dölen et al., 2013; Nardou et al., 2019). However, the presence of Oxtr in axon terminals has not yet been proven. Therefore, our *in vivo* super-resolution imaging could serve as a significant step toward deciphering the subcellular distribution of Oxtr protein in such studies.

The second achievement of the present study was the development of an Oxtr reporter line expressing the bright red fluorescent protein, tdTomato, which is suitable for live imaging. *In vitro* electrophysiological analyses were successfully conducted on tdTomato-positive neurons in our reporter line. Such analyses may not have been possible in the *Oxtr-Venus* line (Yoshida et al., 2009), because expression levels of Venus-positive cells have been reported to largely decline after three weeks (Newmaster et al., 2020). In addition, tdTomato’s red fluorescence could be advantageous when calcium indicators such as the genetically encoded GCaMPs are used, as they mainly contain GFP and emit green fluorescence which overlaps with Venus signals.

Third, we generated both constitutive and inducible Cre drivers for Oxtr-expressing neurons. IRES elements, used in the existing two *Oxtr-IRES-Cre* knock-in lines, have been reported to occasionally cause lower expression of the downstream cistron because of factors such as experimental cell types or cloned gene cassettes (Ibrahimi et al., 2009). Accordingly, we employed the 2A peptide system, which allowed nearly equimolar expression of two cistrons (Trichas et al., 2008; Tang et al., 2009). As we paid much attention to maintain the endogenous Oxtr gene configuration in our knock-in allele, the design of our *Oxtr-T2A-iCre* is indeed different from that of *Oxtr-T2A-Cre-D*, available from The Jackson Laboratory (Daigle et al., 2018). While the latter inserted an exogenous bovine growth hormone poly A signal and ignored the Oxtr’s own endogenous poly A sequence (Extended Data Fig. 1-1*e*), our knock-in mice simply made use of the endogenous poly A signal instead (Fig. 1*b*). Unlike the systematically generated Cre allele as a part of a big project, our iCre knock-in allele was carefully customized for the *Oxtr* locus, which is a promising development for future Oxt studies. In addition, our inducible Cre line was suitably responsive to TMX administration. Although intraperitoneal injection is the most popular TMX administration protocol, it may sometimes be toxic to cause the death of test animals. Therefore, we employed an oral administration protocol (Andersson et al., 2010; Yoshinobu et al., 2021) instead, to ensure the safe and sufficient induction of Cre activity.

TMX is a potent and clinically used selective estrogen receptor modulator that blocks effects of estrogens in breast tissues but facilitates estrogen actions in others such as uterus tissues (Osborne, 1998). Thus, chronic TMX treatment has the possibility to interfere with estrogen function in multiple tissues including the brain. Estrogens are involved in social behavior at multiple levels, from the detection and integration of social information to more complex behavior such as social preference, aggression, and social learning and memory (Ervin et al., 2015). As an example, in the hippocampus, estrogens are locally synthesized and modulate memory-related synaptic plasticity by the rapid nonclassical indirect pathway via synapse-localized estrogen receptors (Mukai et al., 2010; Laredo et al., 2014). We hence need to carefully consider the potential interference by TMX to the behavioral outcomes from Oxtr manipulations in using *Oxtr ^3^*^×^*^HA-iCreERT2^* mice.

Oxt and arginine vasopressin (Avp) are evolutionally related molecules that differ only in two of the 9 aa. Since their receptors also show significant structural homology to one another, the selectivity of Oxt and Avp for their own receptor is not absolute. Considerable receptor crosstalk has been reported to occur among Oxt, Avp, and their receptors (Carter, 2017; Song and Albers, 2018). While exogenously administrated Avp can act on Oxtr in higher concentration and so can do Oxt on Avp receptor 1a (Avpr1a), limited lines of evidence indicate that endogenously released Oxt and Avp can produce functionally significant responses by activating each other’s receptors (Song and Albers, 2018). Based on receptor autoradiography approach with relatively low resolution, Oxtr and Avpr1a appear to show distinct and nonoverlapping expression in the rodent brain (Dumais and Veenema, 2016). However, it remains still unknown whether the localization of Oxtr and Avpr1a is mutually exclusive at the synaptic levels, and thus the possibility of crosstalk following synaptic release of these peptides should rigorously be examined. In this context, our new genetic tools could play a part in clarifying these receptors’ distribution with much higher resolution, deepening our understanding of ligand-receptor interactions for Oxt and Avp. Generation of Avpr1a genome-edited mouse lines might further help precisely visualize its endogenous expression profiles.

Oxt is expected as a potential therapeutic for social deficits represented by autism spectrum disorders. Managing socio-emotional behavior in health and disease to increase resilience to chronic stress has become much more important, especially given the ongoing COVID-19 pandemic (Grinevich and Neumann, 2021). Although the preclinical trials of exogenous Oxt administration via intranasal delivery are currently in progress (Quintana et al., 2021), second-generation strategies to potentiate endogenous Oxt signaling should be preferred in the future (Ford and Young, 2021). Moreover, a fundamental shift from the traditional brain-wide chronic pharmacological intervention to a circuit-specific approach is now anticipated, owing to significant advances in our understanding of the neural circuit mechanisms underlying social behavior in animal models (Ford and Young, 2021). Hence, to establish dependable and effective Oxt-based therapeutic interventions, we need reliable animal models to help uncover the mechanisms underlying Oxt-Oxtr circuitry. Our new genetic tools would play a significant role in meeting this demand and we are going to make the four knock-in lines generated in this study quickly and widely available. This will eventually help deepen our knowledge for the elaborated molecular machinery and functional regions of Oxt-Oxtr actions in our own brain.

## Synthesis

Reviewing Editor: Karyn Frick, University of Wisconsin-Milwaukee Decisions are customarily a result of the Reviewing Editor and the peer reviewers coming together and discussing their recommendations until a consensus is reached. When revisions are invited, a fact-based synthesis statement explaining their decision and outlining what is needed to prepare a revision will be listed below. The following reviewer(s) agreed to reveal their identity: Heather Caldwell, Françoise Muscatelli.

This article reports the creation of 4 novel knock-in mouse lines at the Oxtr-locus, allowing a normal expression of the Oxtr transcript and protein, which is important to maintain since the Oxtr-knock out hetreozygotes present an abnormal phenotype. The reviewers thought that the mouse lines could be reliable and powerful tools that would be very useful to the scientific community. The development of these lines of mice could aid researchers in various aspects of their work, including identifying where oxytocin receptors are located on neurons, the types of neurons on which oxytocin receptors are expressed, and will allow for oxytocin receptor-expressing cells to be identified for electrophysiological work. It was felt that these tools are very likely to advance the field if made available to researchers by opening up a new set of tools for researchers interested in the oxytocin receptor to use and by increasing reproducibility and reliability within the field.

With respect to the manuscript itself, the reviewers found this to be a very nice technical paper. They felt that the manuscript was well-written. The construction of all lines was thought to be well described and the characterization of the expression of the different reporters and Oxtr expression at the cell membrane was considered well done and convincing. That said, the reviewers expressed a few concerns that should be addressed in a revised manuscript.

1. Some of the background information that informed some of the specific choices made by the authors with respect to the lines created is a bit thin in some areas. For instance, while the PA epitope tagging is explained a bit, the HA-tag is only mentioned in passing, and it was not clear why the authors use PA instead of HA. While it is understood that the authors are essentially sharing their “story” about what worked well and what had to be altered experimentally, better clarity in the introduction would help support the presentation of ideas that come up later in the text and decrease confusion.

2. The authors state that these lines will inform our understanding of “Oxt-responsive” neurons, this is also indicated in the title of the article. However, the development of these mouse lines will inform research beyond understanding Oxt-Oxtr signaling since vasopressin can also signal through the Oxtr. Thus, the authors may want to consider broadening their statements about the potential impact of the work.

3. There were some problems with English grammar, and thorough proofreading with a focus on grammar should be done to increase readability.
