# Supplementary figures and images for: Targeting Neurons with Functional Oxytocin Receptors: A Novel Set of Simple Knock-In Mouse Lines for Oxytocin Receptor Visualization and Manipulation
Source: eNeuro. 2022 Feb 14;9(1):ENEURO.0423-21.2022. doi: 10.1523/ENEURO.0423-21.2022 (PMC8856715; doi:10.1523/ENEURO.0423-21.2022)

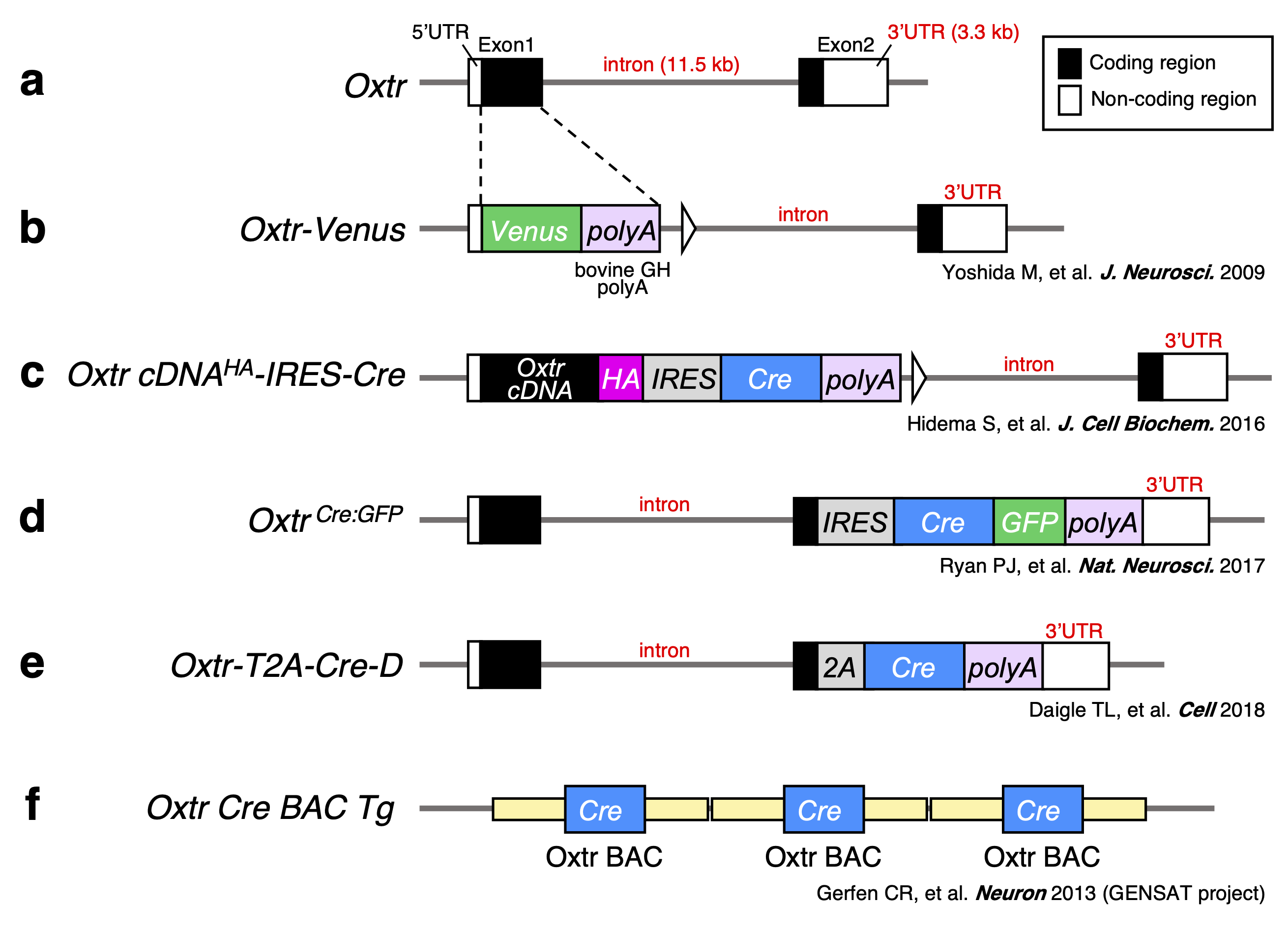

Supplement: Extended Data Figure 1-1 — Already-existing reporter/Cre lines for Oxtr visualization/manipulation. a, Endogenous Oxtr gene configuration is schematically depicted. Oxtr gene has two coding regions shown as solid black bars that are divided by a long intron (11.5 kb). Its 3′ UTR shown as an open bar is also relatively long (3.3 kb). b. Oxtr-Venus knock-in allele (Yoshida et al., 2009). As the 1st coding exon is replaced with Venus sequences followed by bovine growth hormone (GH) polyA signal, this allele lacks the endogenous configuration of intron and 3′ UTR sequences. c, Oxtr cDNAHA-IRES-Cre knock-in allele (Hidema et al., 2016). As is the case with Oxtr-Venus in b, the 1st coding exon is replaced with Oxtr cDNA-HA-IRES-Cre cassette. Although the C-terminally fused HA-tag is detected in uterus tissues, it cannot be detected in brain tissues. d, Oxtr Cre:GFP knock-in allele (Ryan et al., 2017). While the long intron is preserved, the exogenously integrated polyA signal is prioritized over the endogenous Oxtr’s polyA. Exogenous polyA signals might affect stabilities of mRNAs. e, Oxtr-T2A-Cre-D knock-in allele (Daigle et al., 2018). As is the case in d, the exogenously introduced polyA signal is prioritized over the endogenous Oxtr’s polyA. f, Oxtr-Cre BAC transgenic allele (Gerfen et al., 2013). Although bacterial artificial chromosome (BAC) transgenesis is undoubtedly a useful technique, a non-native genomic context via random integration might unexpectedly modify endogenous expression profiles. Download Figure 1-1, TIF file. [file enu-eN-MNT-0423-21-s04.tif]

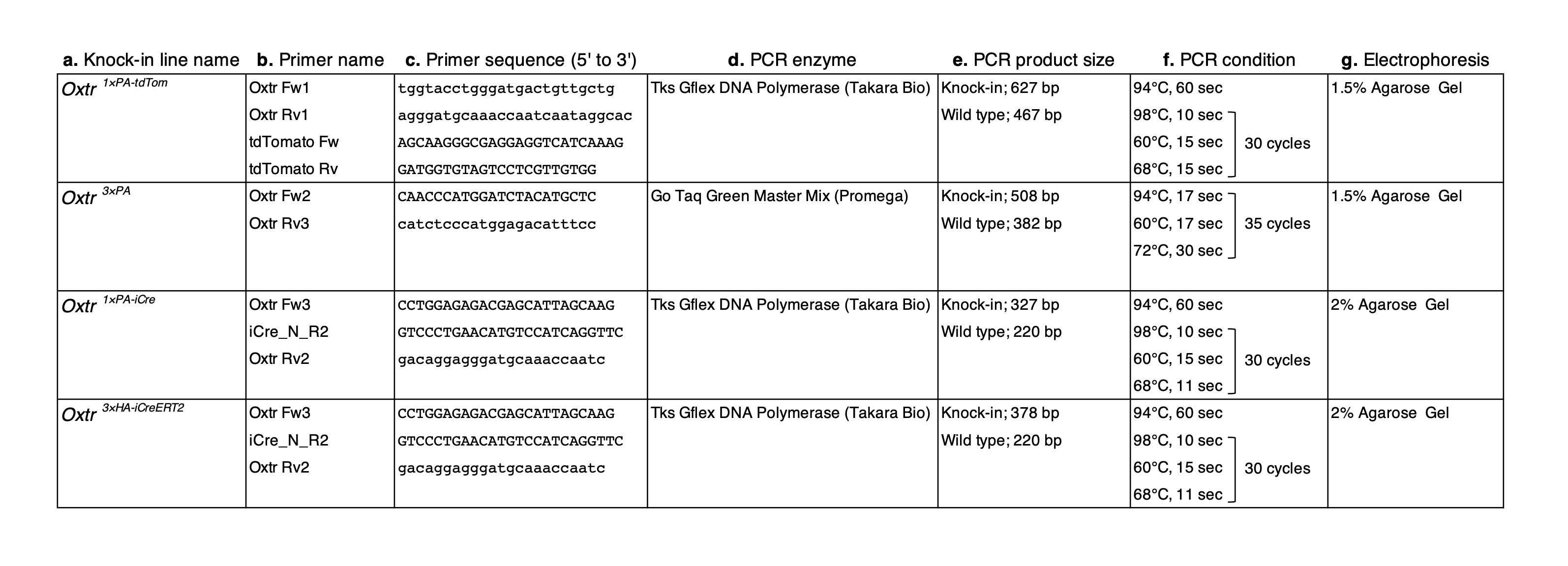

Supplement: Extended Data Figure 1-2 — Genotyping primers and PCR conditions used in this study. a, Names of four knock-in lines generated in this study are listed. b, Genotyping primer names are listed. c, Primer sequences (5′ to 3′) are listed. d, PCR enzymes used for genotyping are listed. e, PCR products’ size for knock-in alleles and wild-type allele are listed. f, PCR conditions for genotyping are listed. g, Electrophoresis conditions are listed. Download Figure 1-2, TIF file. [file enu-eN-MNT-0423-21-s10.tif]

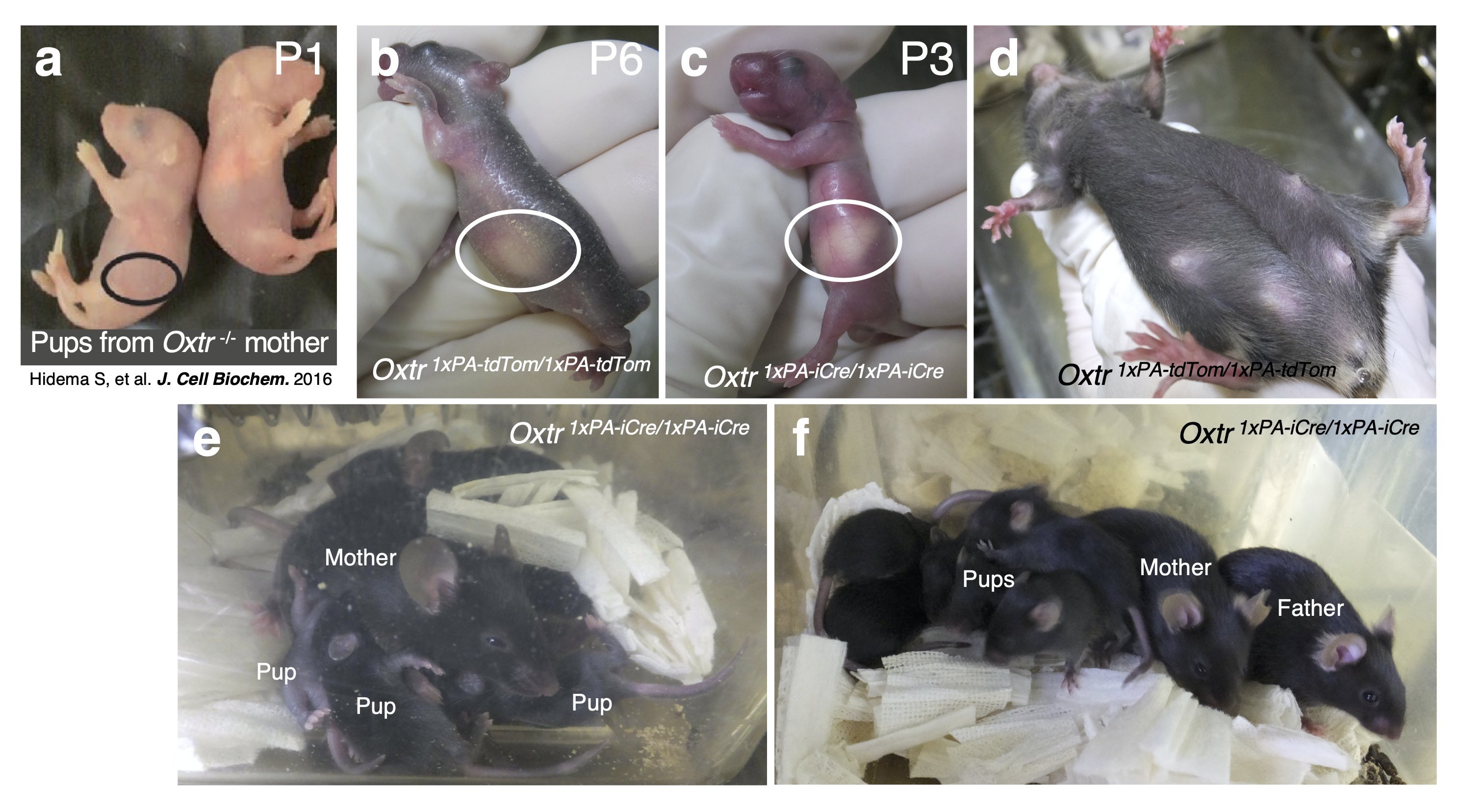

Supplement: Extended Data Figure 2-1 — Homozygous knock-in males and females do not have any problems in their reproductive functions. a, Pups from Oxtr knock-out mothers (Oxtr –/–) has been reported to die soon after birth due to mother’s defects in breast-feeding since Oxt plays essential roles in lactation (Hidema et al., 2016). Pups’ digestive tracts are not filled with milk. b, Digestive tract of a pup from homozygous Oxtr 1×PA-tdTom mother is filled with milk, indicating homozygous mothers have no problems in lactation. c, Digestive tract of a pup from homozygous Oxtr 1×PA-iCre mother is filled with milk, indicating the peripheral Oxt signaling pathway is intact. d, Mammary glands of homozygous Oxtr 1×PA-tdTom mother are well-developed. e, A homozygous Oxtr 1×PA-iCre mother can nurse her pups by lactating. f, A pair of homozygous Oxtr 1×PA-iCre mother and father, and their homozygous progenies. Homozygous knock-in males are fertile. Download Figure 2-1, TIF file. [file enu-eN-MNT-0423-21-s05.tif]

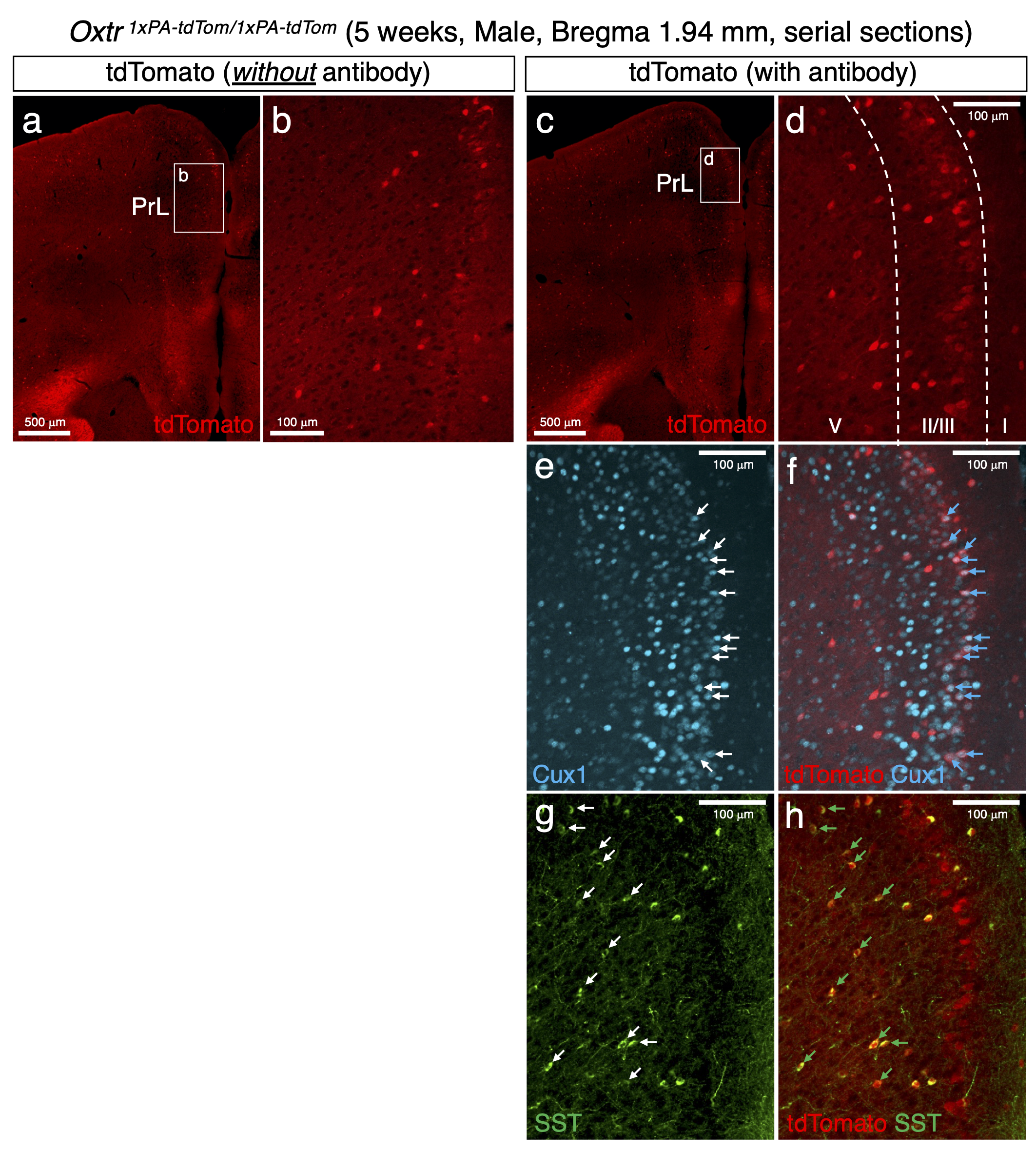

Supplement: Extended Data Figure 3-1 — Characteristics of tdTomato-positive neurons in the PrL. Serial coronal sections of five-week-old homozygous Oxtr 1×PA-tdTom male brain at bregma 1.94 mm are arranged. a, Endogenous tdTomato fluorescence can be detected without immunostaining. b, Enlarged image of the boxed area in a. c, Enhanced tdTomato fluorescent signals are detected with anti-tdTomato antibody. d, Enlarged image of the boxed area in c indicates tdTomato-positive cells reside in both Layer II/III and in Layer V of the PrL. e, f, tdTomato-expressing neurons in Layer II express Cux1, one of the glutamatergic neuron markers. g, h, tdTomato-expressing neurons in Layer V express SST, one of the interneuron markers. Download Figure 3-1, TIF file. [file enu-eN-MNT-0423-21-s06.tif]

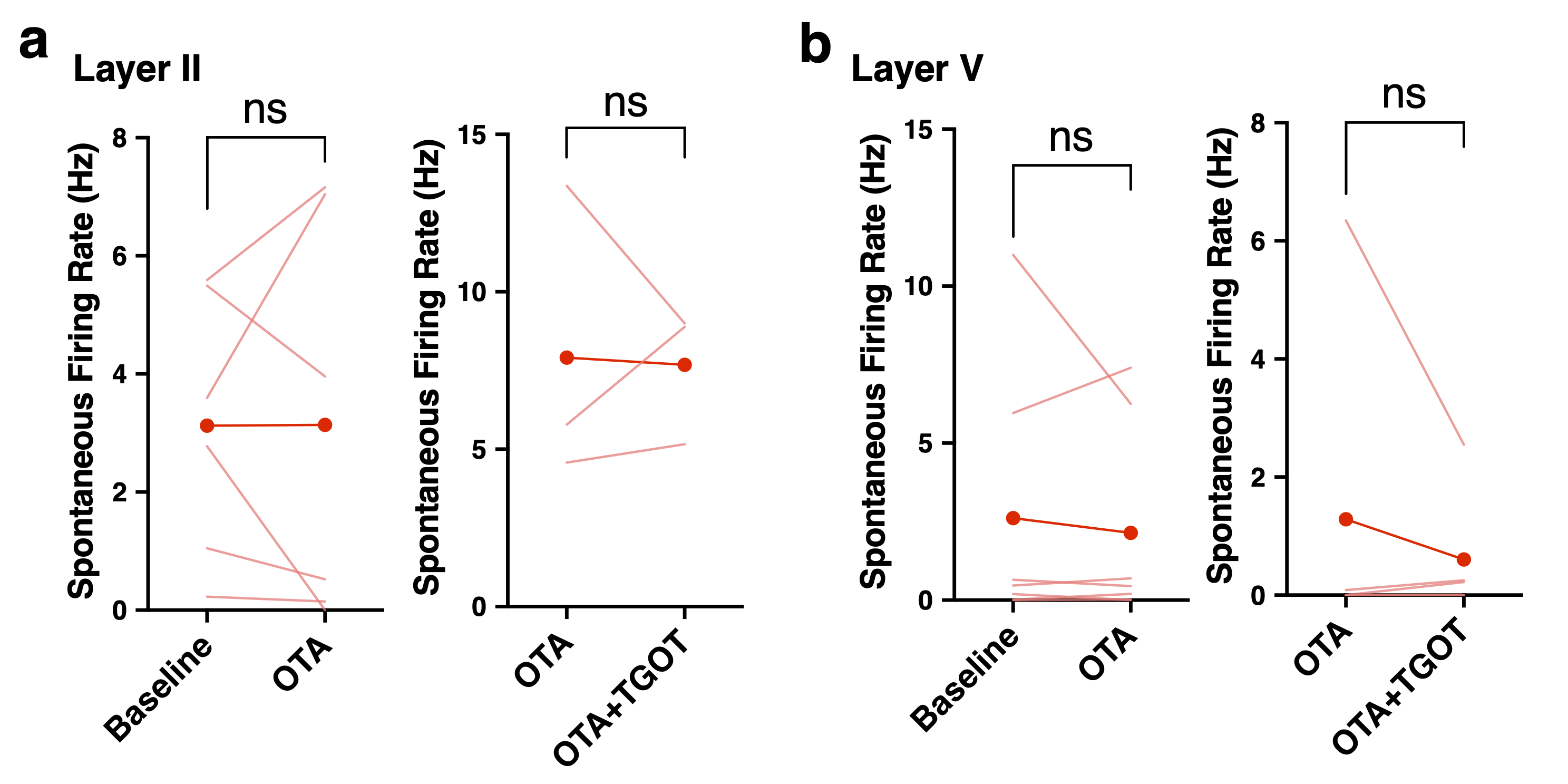

Supplement: Extended Data Figure 3-2 — Blocking effects of Oxtr antagonist, OTA on TGOT-induced increase of spontaneous firing activities in tdTomato-positive neurons. a, Mean spontaneous firing activities of Layer II tdTomato-positive cells in the PrL from Oxtr 1×PA-tdTom homozygous males. Light red lines represent individual neurons’ responses. Red circles with line represent average response. Firing activities remain unchanged in the presence of 1 μm OTA (left panel, n = 6 cells from 4 mice); 1 μm TGOT application in the presence of 1 μm OTA does not increase firing activities of tdTomato-positive cells (right panel, n = 3 cells from 3 mice). ns, not significant. b, Mean spontaneous firing activities of Layer V tdTomato-positive cells in the PrL from Oxtr 1×PA-tdTom homozygous males are arranged. Firing activities remain unchanged the in presence of 1 μM OTA (left panel, n = 7 cells from 6 mice); 1 μm TGOT application in the presence of 1 μm OTA does not affect firing rates of tdTomato-positive cells (right panel, n = 5 cells from 3 mice). Download Figure 3-2, TIF file. [file enu-eN-MNT-0423-21-s07.tif]

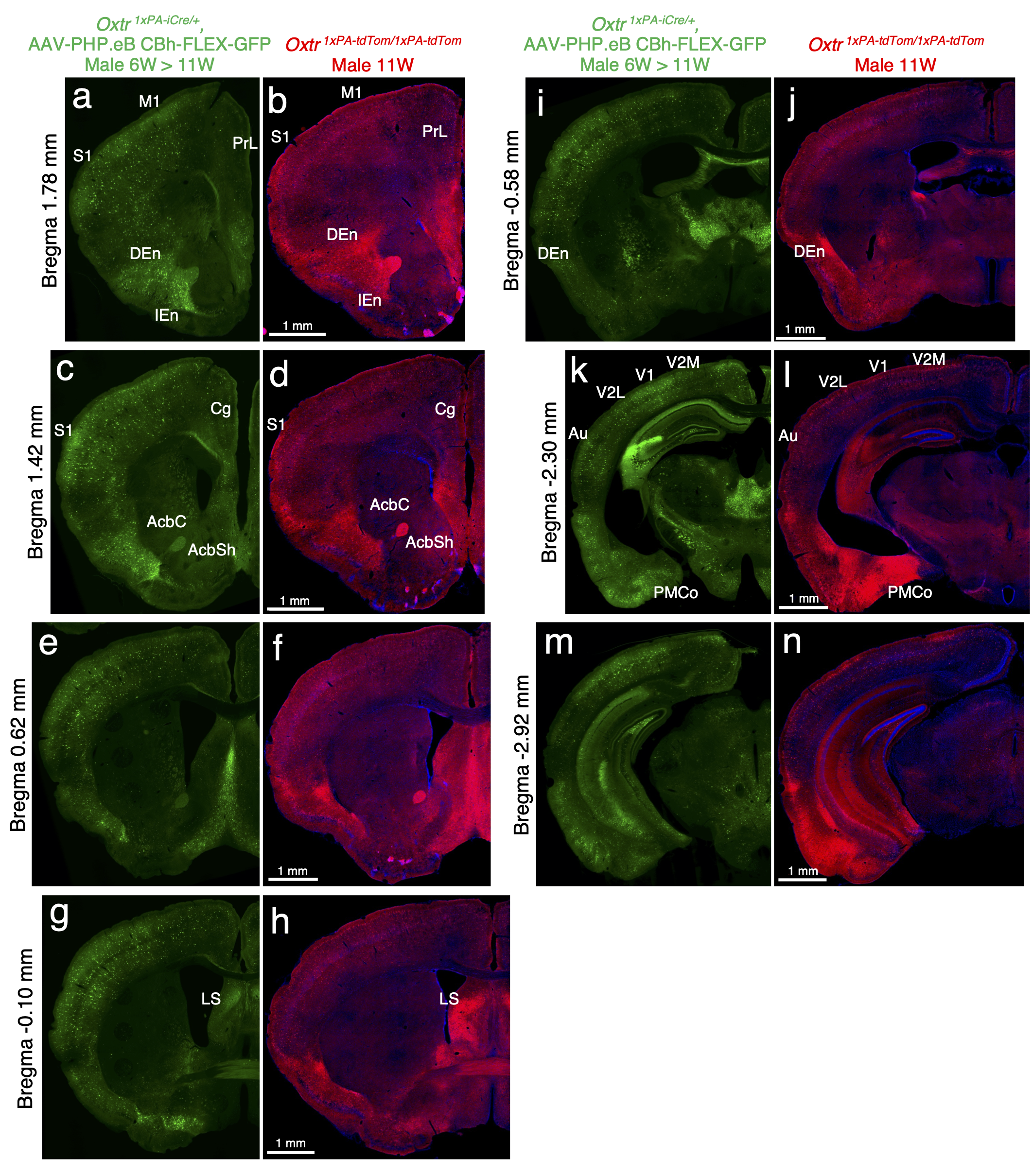

Supplement: Extended Data Figure 4-1 — Atlas of iCre/tdTomato expression profiles. Left side, Expression profiles of GFP in Oxtr 1×PA-iCre heterozygous brain from 11-week-old male to which AAV-PHP.eB CBh_FLEX-GFP vector has been retro-orbitally injected at six weeks. Right side, tdTomato expression profiles in Oxtr 1×PA-tdTom homozygous brain from 11-week-old male. a, b, Bregma 1.78 mm. M1, primary motor cortex; S1, primary somatosensory cortex; DEn, dorsal endopiriform claustrum; IEn, intermediate endopiriform claustrum. c, d, Bregma 1.42 mm. Cg, cingulate cortex; S1, primary somatosensory cortex; AcbC, accumbens nucleus, core; AcbSh, accumbens nucleus, shell. e, f, Bregma 0.62 mm. g, h, Bregma −0.10 mm. LS, lateral septum. i, j, Bregma −0.58 mm. k, l, Bregma −2.30 mm. V2M, secondary visual cortex medial area; V1, primary visual cortex; V2L, secondary visual cortex lateral area; Au, auditory cortex. m, n, Bregma −2.92 mm. Download Figure 4-1, TIF file. [file enu-eN-MNT-0423-21-s08.tif]

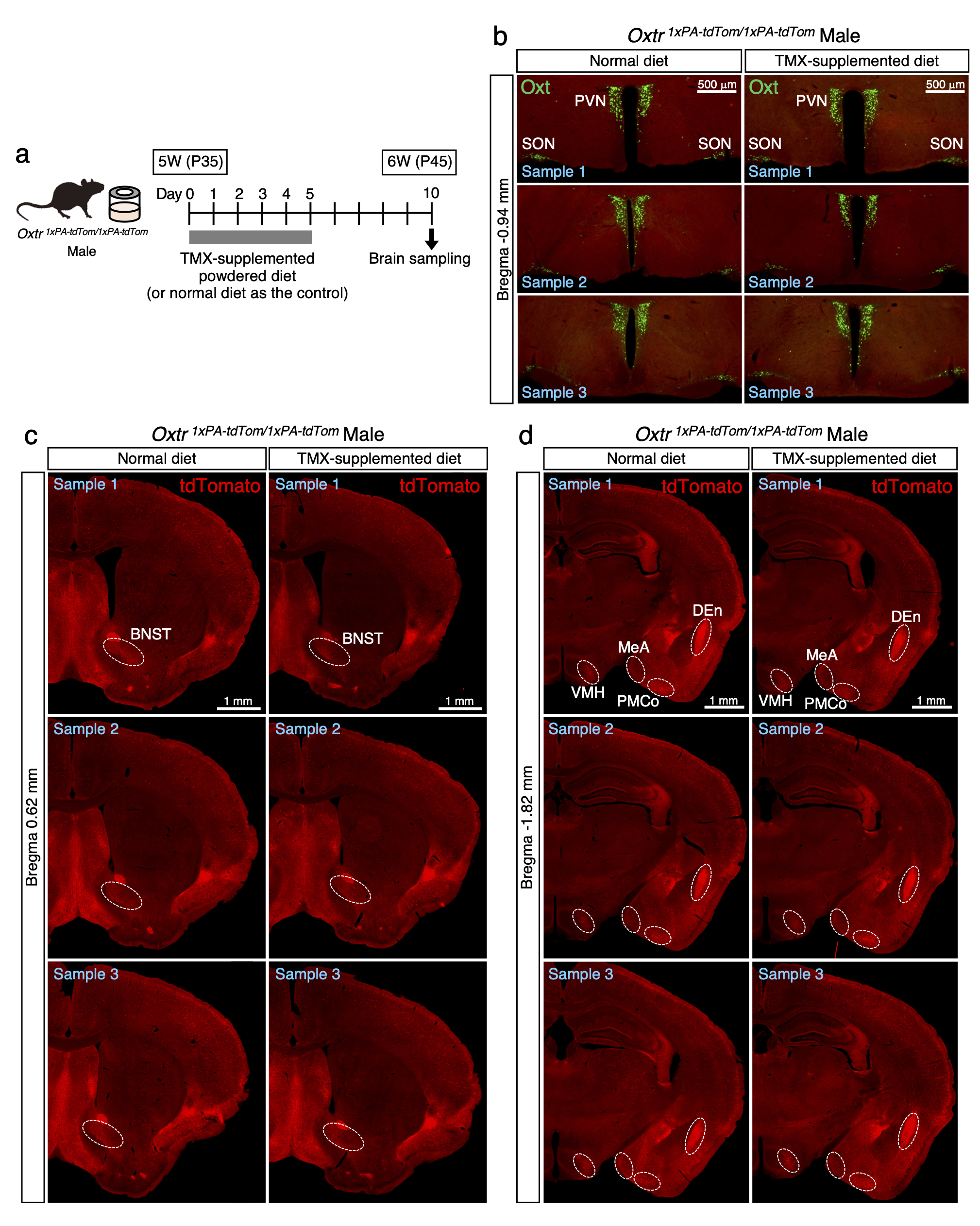

Supplement: Extended Data Figure 5-1 — Five-day TMX treatment has no effect on Oxt/Oxtr expression profiles in male Oxtr 1×PA-tdTom/1×PA-tdTom mice. a, Schematic diagram of experimental protocol. Five-week-old homozygous Oxtr 1×PA-tdTom males can freely access to TMX-supplemented diet from day 0 to day 5 (n = 3). At day 10, brains are sampled for immunostaining. For control experiments, TMX-free normal diet is fed to mice with the same genotype (n = 3). b, Oxt expression profiles in respective Oxtr 1×PA-tdTom/1×PA-tdTom male brain are arranged (bregma −0.94 mm). Left column shows Oxt-expressing neurons in the PVN and SON from normal diet conditions. In right column, 5-d TMX treatment has no effect on Oxt expression profiles in these nuclei. c, tdTomato expression profiles in respective Oxtr 1×PA-tdTom/1×PA-tdTom male brain are arranged (bregma 0.62 mm). In this knock-in line, tdTomato monitors real-time Oxtr expressions. Left column shows tdTomato-expressing cells in the BNST from normal diet conditions. In right column, 5-d TMX treatment has no effect on tdTomato expressions in the BNST. d, tdTomato expression profiles in respective Oxtr 1×PA-tdTom/1×PA-tdTom male brain are arranged (bregma −1.82 mm). Left column shows tdTomato-expressing cells in the VMH, MeA, PMCo, and DEn from normal diet conditions. In right column, 5-d TMX treatment has no effect on tdTomato expressions in these brain areas. Download Figure 5-1, TIF file. [file enu-eN-MNT-0423-21-s11.tif]
